# The impact of community engagement as a public health intervention to support the mental well-being of single mothers and children living under housing insecure conditions – a rapid literature review

**DOI:** 10.1186/s12889-023-16668-7

**Published:** 2023-09-26

**Authors:** Natasha Joseph, Anne-Marie Burn, Joanna Anderson

**Affiliations:** 1 Department of Public Health and Primary Care, University of Cambridge, Cambridge, CB2 0SR UK; 2 Department of Psychiatry, University of Cambridge, Cambridge, CB2 0SZ UK Herchel Smith Building for Brain and Mind Sciences, Forvie Site, Robinson Way,

**Keywords:** Community engagement, Mental health, Mothers, Children, Housing insecurity, Homelessness, Public health interventions

## Abstract

**Background:**

In the UK, the population of homelessness and housing insecurity is increasing among families headed by mothers. The unique stressors of housing insecurity and living in accommodations ill-suited to long-term dwellings increase mental distress for mothers and children. Community engagement interventions present a public health opportunity to alleviate adverse outcomes for vulnerable families.

**Aim:**

To synthesise and evaluate evidence of the impact of community engagement interventions in supporting the mental well-being of mothers and children living under housing insecure conditions. To synthesise the components of community engagement interventions as a public health intervention in alleviating mental well-being and non-health outcomes of mothers and children living under housing insecurity.

**Methods:**

A systematic search of five online bibliographic databases (MEDLINE, EMBASE, PsychINFO, Global Health and Child Development & Adolescent Studies) and grey literature (Carrot2) was conducted in May 2022. Primary studies with community engagement components and housing-insecure single-mother families were included. Intervention data was extracted using the TIDieR checklist and a community engagement keywording tool. The studies’ quality was critically appraised using the MetaQAT framework.

**Results:**

Ten studies meeting inclusion criteria were identified, across two countries (USA & UK). Data from the studies reported positive significant effects for health and personal maternal outcomes in addition to higher positive effects for child health outcomes (e.g., decrease in depression symptoms). Interventions targeting social support and self-efficacy demonstrated potential to improve maternal and child outcomes via the maternal-child relationship. Community engagement at the design, delivery and evaluation intervention stages increased the level of community engagement, however there were tentative links to directly improving mental well-being outcomes.

**Conclusion:**

There is evidence to suggest that community engagement may be applied as an effective intervention in supporting the mental well-being of mothers and children living under housing insecurity. Proposed intervention effectiveness may be achieved via psychosocial pathways such as improved maternal self-efficacy and social support. However, more embedded long-term process evaluations of these interventions are needed to establish maintenance of these observed benefits and to understand to what extent the findings apply to the UK context.

**Supplementary Information:**

The online version contains supplementary material available at 10.1186/s12889-023-16668-7.

## Background

Housing is a recognised social determinant of health [1]. Chronic shortages of affordable housing, high housing costs and stagnating wages contribute to the UK housing crisis and increase the risk of homelessness for families on low incomes [2]. Housing insecurity is related to household expenditure where severe insecurity increases the risk of evictions, the prevalence of temporary accommodation and homelessness [2–4]. Temporary accommodation is offered to at-risk families who seek help from their local authority and are granted by recognising their ‘statutory homeless’ status [5]. In 2021, 1.2 million households were reported to be on the English local authority housing waiting list due to housing insecurity [6] and 96,060 households were living in temporary accommodation in England, including 121,680 dependent children [7]. Current policies are focused on demand-level interventions to address the shortfall in housing, with minimal investment in increasing the housing supply [8]. In 2012, the combination of housing availability and welfare cuts led to a 19% increase in councils providing temporary accommodation to vulnerable households [2]. To meet the rising housing demand, local council spending increased in the private rented sector, reducing the standard of accommodation. Experiences of overcrowding and poor housing conditions increased as hostels, office conversions and bed-and-breakfast hotels are utilised as temporary accommodations [9]. However, the term ‘temporary’ acts as a misnomer as families can live in dwellings for months extending into years as they await to be rehoused in suitable long-term accommodation [9, 10]. The UK charity Shelter, identified 61% of households have spent a year or more living in temporary accommodation, increasing to more than two thirds (68%) of families [11–13]. Further data from 2014 identified over 2000 families with children who have spent between five to ten years living in temporary accommodation in London, an area which has the highest proportion of families living in temporary accommodation in the UK [14]. In 2023, 83,473 children were currently living in temporary accommodation in London which equates to at least one child in every London classroom is homeless on average [15].

### Community engagement

Prolonged exposure to housing ill-suited for a long-term dwelling has a well-established link to adverse mental well-being and poor child development [16]. Parents living in temporary accommodation have poorer mental health outcomes such as depression and anxiety compared to housing-secure parents [17]. In addition, the parental-child relationship model elucidates the translation of poor parental mental well-being to adverse childhood experiences as shown in poor educational attainment, mental health and behavioural outcomes [3, 18]. Families headed by single mothers form the majority of families living in temporary accommodation in the UK [19]. Given the chronic stressors and mental well-being disparities, therein lies the need to adapt public health interventions for single mothers experiencing chronic housing insecurity.

NICE guidelines recognise public health interventions such as area-based initiatives and urban regeneration programmes to reduce health inequalities [20]. The intention is to tackle the socioeconomic determinants of health through investments that reduce deprivation such as funding education, income, housing, and employment initiatives. However, such macro-level interventions encounter pitfalls such as poor assessment criteria for measuring individual impacts such as health outcomes [21, 22]. Furthermore, area-based initiatives may fail to equitably incorporate dialogue between marginalised communities and decision-makers in institutions owing to asymmetric power structures, cultural differences, and poor relationships with statutory organisations [20]. Consequently, the barriers culminate to prevent the development of contextually suitable interventions for the local population [23, 24].

Community engagement is an umbrella term that describes a *‘range of approaches aimed to maximise the involvement of local communities in local initiatives to improve their health and well-being and reduce health inequalities’* ([20] p 11–12). The operationalisation of community engagement into practice can take multiple forms of activities with varying levels of member involvement: information-giving, consultation, joint decision-making, collaboration and supporting independent community interest through empowerment [25]. This paper will utilise the conceptual framework put forth by O’Mara-Eves et al. (2013) where community engagement is conceptualised within health interventions through a dynamic framework that incorporates the processes involved with community engagement and omits unidirectional forms of engagement such as information-giving in favour of bidirectional engagement [26].

A rapid review was carried out to synthesise and evaluate evidence of community engagement programmes in supporting the mental well-being of children and mothers living under housing insecurity. In addition, the review aimed to evaluate the effectiveness of interventions and the components of the intervention involved in alleviating poor mental well-being outcomes for single families.

## Methods

### Search strategy

The report followed the Preferred Reporting Items for Systematic Reviews and Meta-Analyses (PRISMA) guidelines [27]. A comprehensive search of both peer- and non-peer-reviewed articles from online bibliographic databases and grey literature search using relevant MeSH words or subheadings of keywords was conducted in May 2022.

Studies of community engagement as a health intervention for the support of mental well-being among children and mothers experiencing housing insecurity were identified from five bibliographic databases (MEDLINE, EMBASE, PsychINFO, Global Health and Child Development & Adolescent Studies).

Grey literature searches for empirical primary data were conducted using a hand-search reference list of included studies and relevant reviews. To maintain robustness, the majority of grey literature searches were conducted on Carrot2 Clustering Engine (https://search.carrot2.org/). The search engine was the preferred domain due to the higher reproducibility of the search strategy in comparison to Google [28].

The search strategy related to the population, outcome, and intervention (Table 1). Full-search strategies are available in Appendix Tables 1–5. The search strategy aimed to capture the broad spectrum of terms that fall under umbrella phrases such as community engagement and mental wellbeing by using synonyms and proximity searching.

**Table 1:** Population, intervention and outcomes characteristics and search terms

Characteristic	Search Terms
Mother and/or child	wom#n or girl* or female* or mother* or famil* or care* or maternal or antenatal or bab* or prenatal or playgroup* or birth* or child* or infant* or neonat* or newborn* or pregnan* or postnatal
Housing insecurity	homeless* or hostel* or shelter* or statutory service* or evict* or crowding or overcrowd* or crowd* or public housing or 'housing tenure' or dwelling
Community engagement	commun* adj3 (engag* or organi#ing or organi* or collab* or advocacy or group* or class* or circle* or club* or committee or facilitat* or meeting* or program* or participant or stakeholder
Mental wellbeing	(mental or emotional or psycho*) adj2 (health or wellbeing or well-being or ill* or disorder* or condition* or problem* or difficult*)) or self-efficacy or self-esteem or self-worth or (self and (efficacy or esteem or worth))

Housing insecurity has no clear definition but is characterised as a spectrum ranging from no access to housing of reasonable quality to complete access to housing of reasonable quality in absence of threats (e.g. financial precarity) [29]. Housing insecure inclusion ranges from homeless shelters, bed-and-breakfast, sofa surfing, temporary accommodation, and characteristics such as overcrowding.

Community engagement is defined as ‘involving communities in decision-making and the planning, design, governance and/or delivery of services’ ([30] p. 11). Approaches include but are not limited to healthcare forums, service user networks, peer-led interventions or volunteering [26].

### Inclusion and exclusion criteria

The inclusion criteria are outlined in Table 2. Studies were selected based on the PICO framework (population, intervention, comparator and outcomes) [31]. Only studies published in English language were included and there was no limit placed on the publication date. Focusing on papers written in English enabled the review to identify countries with similar definitions of homelessness to aid interpretation [32]. Non-peer-reviewed literature was searched to include interventions conducted in non-academic institutions such as charity websites.

**Table 2:** Final eligibility for study inclusion

	Inclusion criteria	Exclusion criteria
Population	Mothers with at least one child under 18, pregnant mothers and / or children under 18 who experience housing insecurity such as shelters, bed-and-breakfast, hostels, hotels, sofa surfing, transitional housing	No explicit mention of housing insecurity among children and/or mothers
Intervention	Community engagement such as community consultation, collaboration, service user networks, community-based case management, parental skill training, health-care forums	No mention of community engagement, only inclusion of information provision
Comparator	No comparator or service as usual	None
Outcomes	Related to women and children’s mental well-being such as stress, emotional distress, social support, depression, anxiety, and child behaviour	Service-related outcomes such as adherence to services without inclusion of psycho-social measures
Study design	All primary studies such as quantitative, quasi-experimental studies (e.g., pre-and-post studies), non-randomised control trials, randomised control trials, mix method studies, case–control studies	Essays, theory papers, reviews, think pieces
Location	Worldwide	None
Language	English	Non-English

### Screening and selection

All retrieved titles and abstracts were reviewed by one reviewer (NJ) with a second reviewer independently screening 12% of eligible studies based on the inclusion and exclusion criteria (JA). The interrater reliability was deemed to be in fair agreement (Cohen’s Kappa = 0.31) for the title and abstract screenings [33]. The third reviewer (A-MB) independently resolved conflicts with planned discussions. Full texts of all eligible articles were retrieved and screened by one reviewer (NJ) using the eligibility criteria and excluded with reason.

### Data extraction

Data were extracted based on study characteristics (study aims, sample, methods, outcomes). The intervention characteristics (e.g., study context, intervention reason, intervention details, location of intervention, intervention deliverer) were extracted using the TIDieR checklist to standardise the reporting of the intervention [34, 35] (Appendix Table 10). Outcomes for mothers and children are extracted from O’Mara-Eves et al. (2013) keywording tool ([26] p. 181–189) which categorises the type of intervention based on health, community, personal and process outcomes with identification of positive, non-significant and negative findings from outcomes (Appendix Table 13). Data on the community engagement components were extracted using the tool developed by O’Mara-Eves et al. (2013) Keywording tool ([26] p. 181–189). Data was analysed by one reviewer (NJ) and verified by a second reviewer (JA).

### Data analysis

Due to the heterogeneity of the papers, subgroup analysis was used to explore the impact of interventions across mothers and children respectively. Post-extraction, the reported outcomes were analysed with descriptive statistics. In addition, the heterogeneity of the populations across the papers was organised using the O’Mara-Eves et al. (2013) Keywording tool ([26] p. 181–189) to capture population characteristics such as ethnicity, social economic position, place of residence, education, employment status, gender, marital status and age (Appendix Table 14).

### Critical appraisal

The critical appraisal of the papers was evaluated using the Public Health Ontario Meta-tool for Quality Appraisal (MetaQAT) [36]. MetaQAT framework includes four domains: reliability, relevancy, validity, and applicability in addition to the study-specific appraisal tool (Appendix Tables 6–8). MetaQAT was deemed the most appropriate tool as it provides a systematic and rigorous approach to assessing the broad spectrum of research designs. Furthermore, the tool is contextually relevant to the public health field and overcomes the limitations of critical appraisal tools designed for clinical medicine [37].

## Results

**Fig. 1:**
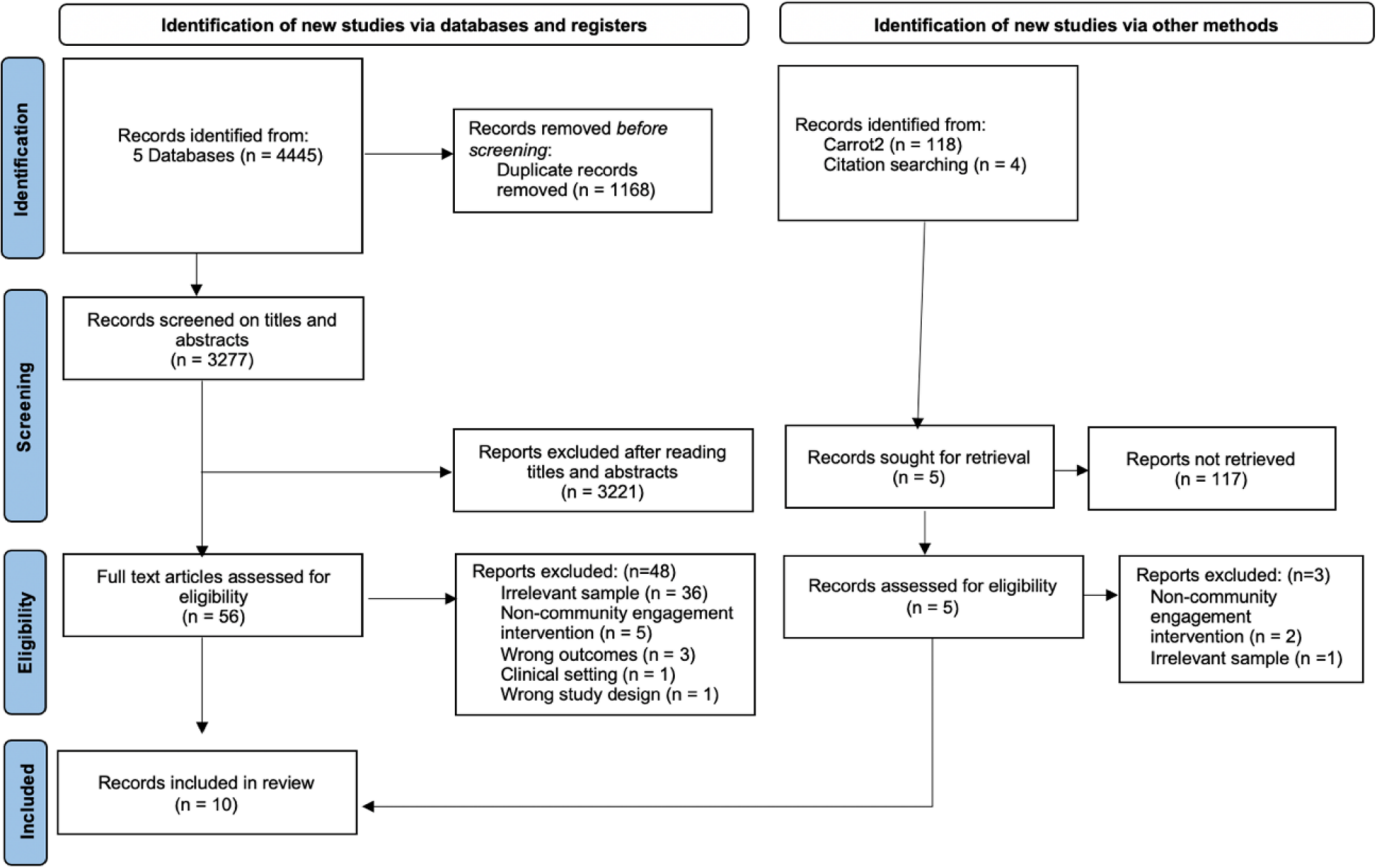
PRISMA Flow diagram of the study selection process

The electronic bibliographic database search resulted in 3277 articles following the removal of 1168 duplicates. The screening phase consisted of title and abstract screening where 3277 studies were screened, and 56 studies were screened at the full-text level. The grey literature search resulted in 121 records and after the screening, one paper contributed to the total number of included studies (*n* = 10). Non-published grey literature was not identified from the search. Figure 1 illustrates the study selection process using the PRISMA guidelines.

Of the 10 studies included in the review, eight were conducted in the United States, and two in the United Kingdom. The studies used various study designs, including observational case–control (*n* = 4), randomised control trial (*n* = 3) and quasi-experimental (*n* = 3). The characteristics of included studies are summarised in Table 3. Two studies had no comparator groups based on the quasi-experimental study designs. Eight studies contained a comparator such as service/treatment/case as usual (*n* = 5) or alternative comparable group (*n* = 3). The comparable sample group were used to compare populations to identify significant differences in health needs (e.g., between low-income families and families in shelters) or whether the intervention required greater adaptation to suit the target population.

**Table 3:** Summary characteristics of included studies (*n* = 10)

Criteria	Characteristics	No. studies
Year	2001–2010	4
	After 2010	6
Country	US	8
	UK	2
Population	Mothers experiencing housing insecurity / homelessness only	4
	Children experiencing housing insecurity / homelessness only	1
	Both	5
Study design	Randomised control trial	3
	Nonrandomised control trial	0
	Quasi-experimental	3
	Observational (case–control)	4
Comparator	No comparator	2
	Comparator (service-as-usual, different sample group)	8
Outcomes (a)	*Maternal outcomes*	
	Community outcomes: Social support, social capital	3
	Health outcomes: mental wellbeing, mental health service use	7
	Personal outcomes: Self-efficacy, self-confidence, self-esteem	4
	*Child outcomes*	
	Health outcomes (child behaviours, mental health service use)	6
Number of outcome measurements^a^	Pre- and post-intervention	4
	One further timepoint	0
	Two further timepoints	3
	Post intervention only	4
Length of follow-up period (including additional timepoints)	1–3 months	5
	4–6 months	2
	7–9 months	1
	10–12 months	1
	> 12 months	2
	No information provided in study	2

^a^More than one outcome could be measured for each intervention and so total does not add up to 10 included studies

Sample sizes were relatively small varying from 15 to 267 participants, with four studies focusing on mothers experiencing housing insecurity, one focusing on children experiencing housing insecurity, and five focusing on both mothers and children. All studies with children as participants addressed health outcomes such as child behaviour and mental health service use (*n* = 5). All studies with mothers as participants included three types of outcomes: health outcomes such as maternal mental well-being (*n* = 7), personal outcomes such as self-efficacy (*n* = 4) or community outcomes such as social support / social capital (*n* = 2). The categorisation of outcomes was based on the O’Mara-Eves et al., (2013) data extraction tool ([26] p. 191–195).

### Quality of studies

The critical appraisal of included studies is summarised in Table 4 and provided in detail in Appendix Tables 6–8. The MetaQAT tool identified the quality of included studies based on relevancy, reliability, validity, and applicability to the public health context [36]. The majority of papers had a high quality of relevancy (*n* = 9) and validity (*n* = 9). Five studies had the highest level of reliability which included the reporting of consent and ethical approval from a university board. The distinction between moderate to high reliability was the presence of board ethical approval in addition to participant consent.

**Table 4:** MetaQAT rating of included studies (*n* = 10)

Study	MetaQAT assessment components			
	Relevancy	Reliability	Validity	Applicability
Abell et al. (2009) [38]	High	High	High	High
Bradley et al. (2020) [39]	High	High	High	High
Brown et al. (2020) [40]	High	High	High	High
Gewirtz et al. (2015) [41]	High	Moderate	High	High
Lee et al. (2010) [42]	High	High	High	High
McWhirter (2006) [43]	Moderate	Moderate	High	Low
Nabors et al. (2004) [44]	High	Moderate	High	Moderate
Samuels et al. (2015) [45]	High	High	High	High
Weinreb et al. (2016) [46]	High	Moderate	High	High
Zhang, Limaye & Means (2021) [47]	High	Low	Low	Low

### Components of community engagement characteristics

Community engagement health interventions contain multiple and complex components to adapt to the needs of the target population. The components focus on the level of community engagement and contextual factors of community engagement interventions. Table 5 outlines the labels of community engagement based on O’Mara-Eves et al., (2013) extraction tool and identified community organisation label as the most frequent strategy followed by peer strategy. A full break down of community engagement strategy label by study can be found in Appendix Table 15.

**Table 5:** Labels for community engagement strategies

Community engagement strategy label	Total
Community action/support, community mobilisation/involvement/engagement/participation	1
Community organisations – developing new and existing services	8
Community coalition, community partnership, community task force	1
Any peer strategy (e.g., peer counselling, peer education, peer leaders, peer leadership, role models, peer support)	3
Non-peer health advocacy (e.g., lay health workers, community health)	2
Social networks (explicit use of the term)	2

Note that total is greater than 10 as some interventions can be described by more than one label

#### Level of community engagement

The extraction of community engagement characteristics aims to identify the level of community involvement within stages of the intervention such as the design and planning of the intervention, the delivery, and the intervention evaluation (Table 6). Categorising involvement was based on O’Mara-Eves et al., (2013) extraction tool which identified six levels of involvement: leading, collaborating, consulted, informed, and not involved (or not clear). Two studies maintained high levels of community engagement at each stage of the intervention [39, 40]. The majority of studies relied on informing-based delivery of community engagement. Low levels of community engagement are involved in the designing and planning phase of the intervention with only two UK studies providing evidence of community engagement at preliminary stages [39, 40].

**Table 6:** Community engagement components based on the stage of the intervention

Study	Community engagement component of intervention stage		
	Design / planning	Delivery	Evaluation
**Abell et al. (2009)** [38]	Not involved/unclear	Informed	Informed
**Bradley et al. (2020)** [39]	Consulted	Collaborating	Consulted
**Brown et al. (2020)** [40]	Collaborating	Leading	Consulted
**Gewirtz et al. (2015)** [41]	Not involved/unclear	Informed	Consulted
**Lee et al. (2010)** [42]	Not involved/unclear	Informed	Not involved/unclear
**McWhirter (2006)** [43]	Not involved/unclear	Informed	Not involved/unclear
**Nabors et al. (2004)** [44]	Not involved/unclear	Informed	Consulted
**Samuels et al. (2015)** [45]	Not involved/unclear	Informed	Informed
**Weinreb et al. (2016)** [46]	Not involved/unclear	Informed	Informed
**Zhang, Limaye & Means (2021)** [47]	Not involved/unclear	Informed	Not involved/unclear

#### Contextual components of community engagement

Various types of deliverers were involved in implementing the intervention such as health professionals, community workers, counsellors, social workers, and peers such as mothers experiencing housing insecurity (Table 7). Interventions were conducted in a range of settings such as shelters, churches, clinics, and community centres. Two study interventions took place in housing agencies which are supportive sites with a range of services to support families experiencing housing insecurity and act as an intermediary between temporary accommodation and permanent housing (Table 7). The studies adopted various intervention strategies to improve maternal and child outcomes. The most frequent intervention strategy included providing social support (20%), education (12%) and access to services (12%) (Table 8). Four studies provided training, three to participants [39, 40, 42] and one to interventionists [46]. Volunteers were described in the context of providing childcare so that mothers could fully participate in the intervention. The intervention duration ranged from 1 to 30 months (Appendix Table 9).

**Table 7:** Intervention deliverer categories and location categories

Who delivered the intervention?	Total^a^
Volunteers	2
Peer (parents/mothers experiencing housing insecurity)	3
Health professional	3
Community worker	2
Researcher	1
Counsellor/therapist	3
Social workers	2
Where was the intervention delivered?	Total^a^
Temporary accommodation (shelter/hostel)	3
Housing agency	2
School	1
Outdoor setting (e.g., camp)	2
Church venue	1
Community centre	3

^a^More than one outcome could be measured for each intervention and so the total does not add up to 10 studies

**Table 8:** Intervention strategies of included studies

Intervention Strategy	**Frequency**	**Percentage**
Activities	3	7%
Advice	1	2%
Clinical treatment	2	5%
Counselling	1	2%
Education	5	12%
Medical screening	1	2%
Needs assessment (not screening)	2	5%
Physical activity	1	2%
Professional training	1	2%
Resource access	3	7%
Service access	5	12%
Skill development training	3	7%
Social support	8	20%

*Note*: More than one intervention strategy could be selected for each intervention and so percentages do not sum to 100%

### Process evaluation of intervention

Eight out of the 10 studies contained a process evaluation of the intervention. Table 9 summarises the process evaluation across the included studies. The process evaluation identifies the nature of delivery and adherence to the intervention to understand the effectiveness of the intervention for the target population. Four studies analysed the participants’ fidelity to the intervention. Four studies analysed the acceptability and satisfaction of the intervention. Bradley et al. (2020) adopted a mixture of questionnaires and qualitative interviews to determine the acceptability and feasibility of the intervention. Three studies identified training provided to interventionists (care managers and primary care physicians). No study provided an evaluation of cost-effectiveness. Seven studies provided information about the sources of funding from charities (*n* = 1) and research/state grants (*n* = 6).

**Table 9:** Processes evaluation of included studies (*n* = 10)

Study	Process variable				
	Acceptability / Satisfaction	Accessibility / Feasibility	Fidelity	Quality of programme materials	Interventionist skills training
Abell et al. (2009) [38]					x
Bradley et al. (2020) [39]	x	x		x	
Brown et al. (2020) [40]	x		x		
Gewirtz et al. (2015) [41]			x		
Lee et al. (2010) [42]			x		
McWhirter (2006) [43]					
Nabors et al. (2004) [44]	x	x			
Samuels et al. (2015) [45]			x		x
Weinreb et al. (2016) [46]					x
Zhang, Limaye & Means (2021) [47]					
Total	3	2	4	1	3

Nabors et al. (2004) evaluated acceptability and accessibility process variables using questionnaires given to children and teachers respectively [44]. Gewirtz et al. (2015) identified fidelity to intervention using attrition analysis of the participant follow-ups and identified no significant differences between the intervention group and control [41]. Fidelity to community, counselling or medical services was identified using a structured review in Lee et al. (2010) [42]. Brown et al. (2020) reported high satisfaction rates with the implementation and planning of an intervention, but lower ratings were given for participant involvement in the intervention planning [40]. Bradley et al. (2020) provided the perspectives of mothers living in hostels on the appropriateness of the adapted intervention content and feasibility within the environment [39].

### Effectiveness of community engagement intervention

The analysis of the 10 studies identified two broad intervention categories: community-based models (*n* = 6) and peer-led models (*n* = 4). The community-based models are characterised by a trained professional as the lead, where the intervention pathway is facilitated through the relationship between a trained professional and the mother and/or child (Table 10). The peer-based models focus on the relationship between different mothers and/or children experiencing housing insecurity but are not solely led by professionals or facilitated through the professional-mother/child relationship (Table 11). Improvements to social support were observed across the intervention groups under community-based studies [38, 43]. Improvements in attendance to appointments with a health professional were observed [44, 46, 47]. In addition, reductions in mental distress conditions such as depression were observed in two studies, however the reduction could not be attributed to the community-based interventions as no difference was identified upon control-intervention group comparison [45, 46]. Studies under the peer-based models observed social support outcomes being impacted by the level of housing insecurity where no change was observed [39, 40]. Bradley et al. (2020) operationalised social support into seven domains and two total network analysis in a pre-post study design [39]. Domains affected by resource availability and physical environment such as material aid, socialising, tangible assistance identified no changes from baseline and 6-month follow-up [39]. However, the intervention identified positive changes in domains relating to social need such as network size for advice/information, network for pregnancy/childcare support, total network satisfaction and intimate interaction [39].

**Table 10:** Intervention of community-based models with professional lead and outcomes of included studies

Study	Study design	Intervention details (Name of intervention, components, and intervention strategy)	Outcome
Abell et al. (2009) [38]	Between-subject design (controlled study)	**The Community Case Management (CCM) Intervention** The CCM intervention addresses individual, family, and neighbourhood stressors that may impair family functioning and also diminish their capacity to move toward permanent housing in these vulnerable families *Strategy:* Social support, service access, Resource access (food assistance),	**Child outcomes** Children externalising behaviour improved in the intervention group compared to the control group (*p* = 0.01, Cohen's d = 0.75 large effect size) No difference in internalising behaviours (Cohen’s d not reported) **Family outcomes** Social support increased in intervention group Large effect size d = 0.833, *p* = 0.017, 95% CI = NR Improved family relationship over control group: Large effect size d = 0.952, *p* = 0.0135, 95% CI = NR) Communication subscale Large effect size d = 1.066, *p* = 0.01, 95% CI = NR Unity dimension Large effect size d = 0.952 *p* = 0.0135, 95% CI = NR
McWhirter (2006) [43]	Between-subject design (controlled study)	**Community-based group therapy** Combination of cognitive behavioural and gestalt therapeutic techniques that focused around a curriculum on (a) exploring personal belief systems, especially concerning difficult experiences; (b) understanding the various forms of abuse; (c) understanding and expressing feelings; (d) recognising healthy relationships; (e) and finding healthy ways to cope with stress Strategy: Counselling, social support	**Child outcomes: N/A** **Maternal outcomes:** - Social support variables: Overall increase between both groups in social network size and social isolation with a significant increase among the comparison group (women in transition). Calculated using one-factor ANOVA Social Network Size, F (1, 53) ¼ .02, *p* > .01, Social Isolation, F (1,66) ¼ .43, *p* > .01 - Self-efficacy greater increase in group among women who were living in homeless shelters (intervention) than women in transition group (alternative comparison group i.e. individual educational mentorship group) [Self-efficacy F (1,65) ¼ .49, *p* > .01.] - Decreases in financial stress following participation in both groups Financial Stress, [F (1,53) ¼ .19, *p* > .01] -Family conflict [Family Conflict, F (1,64) ¼ .00, *p* > .01] - No effect on family bonding and / or family conflict for either interventions [Family Bonding F (1,59) ¼ .26, *p* > .01]
Nabors et al. (2004) [44]	Between-subject design (controlled study)	**School Mental Health Program & Empowerment Zone Project** - Small group prevention activities and individual counselling session for children experiencing homelessness during recreation period in afternoons - 10 classroom sessions delivering health promotion on violence prevention, stress management, conflict resolution, risks associated with smoking and drug use, and techniques for improving emotional expression and social skills, enhancing self-esteem, using relaxation techniques, and discussed ways to be on-task, get work done, and behave appropriately in the classroom. Health promotion topics included dental and physical hygiene, learning about germs and colds and the importance of hand washing, and healthy eating and exercise habits *Strategy:* *Education, activities (prevention activities for physical and mental health problems), counselling, advice (on enhancing self-esteem *via* relaxation techniques), needs assessment*	**Child outcomes:** 24 girls and 21 boys completed survey (mean age 7 years and 2 months) - Low income children reported seeing the same doctor on a routine bases whereas only 17% of children experiencing homelessness reported seeing a doctor regularly [Chi-squared test, χ^2^(1) = 8.031, *p* ≤ .01] - Children from low-income families reported receiving more medical services than children experiencing homelessness [Chi-squared test, χ^2^(1) = 3.994, *p* ≤ .05, t = 2.01, *p* ≤ .05] - Children experiencing homelessness reported seeing a counsellor (66.7%) than children from low-income families (7.4%) [Chi-squared test, χ^2^(1) = 17.696, *p* ≤ .01.] - No measurements directly on mental health / other wellbeing indicators **Maternal outcomes**: N/A
Samuels et al. (2015) [45]	Between-subject design (controlled study)	**Family Critical Time Intervention protocol:** Community based case management under three phases: *Transition to Community, Try-Out & Transfer to Care* - Intervention group received continuous case-management services from single worker with CTI training, - Managers had low-caseloads (< 12) in comparison to service as usual (50:1 with high turnover rate among caseworkers) - Low threshold for housing readiness for intervention group than service-as-usual meaning families had more immediate access to transitional housing than service as usual group Strategy: *Social support, Service (employment, child support, family children's services, medical/home care and temporary financial services) and resource access (housing)* *support mothers with children for 9-month period as they transition from homeless shelters to affordable housing*	**Child outcomes: N/A** **Maternal outcomes:** Between baseline and 15-month assessments, mothers reported a 9-point decrease, on average, in standard Global Severity Index scores. The decrease brought the majority of mothers into the normal range of mental health relative to the general adult population No significant difference in mental health and rate of decline between the intervention, Family Critical Time Intervention (FCTI) and control group At 9 month follow up mothers with elevated symptoms declined by 35% in intervention and 35% in control group FCTI intervention group transitioned into permanent housing faster than service-as-usual group however this did not correspond to improvements in maternal mental health
Weinreb et al. (2016) [46]	Between-subject design (controlled study)	***Integrated Care Model for Homeless Mothers*** Collaborative care model adapted to the needs of homeless mothers which included engagement interview with mothers via care manager, providing basic needs (food stamps, clothing for children, obtaining public assistance) and addressing mental health comorbidities *Strategy:* Medical screening, *Professional training for staff members and access to resources and services (e.g., food stamps, clothing, diapers, obtaining public assistance)*	**Child outcomes: N/A** **Maternal outcomes:** Overall significant deduction in depression score for all women by the end of the follow up At 6 months follow up, women in the intervention group had a high per proportion of over 50% improvement in depression symptoms (intervention: 30%, usual care: 5.9%, *P* = .07) Women in the intervention group also had significantly more primary care physician (PCP) and care manager visits at both follow ups: *3-month* PCP visits being two or more—intervention: 74.3%, usual care: 53.3%, *P* = .009; Care manager visits being two or more – intervention: 91.4%, usual care: 26.7%, *P* < .001 *6-month* PCP visits being two or more—intervention: 46.7%, usual care: 23.5%, *P* = .003 Care manager visits being two or more – intervention 70%, usual care: 17.7%, *P* = .001, Medication prescription for depression – intervention: 73.3%, usual care: 5.9%, *P* ≤ .001 No difference between intervention and usual care groups when assessing anxiety, mental or physical health functioning, patient reactions assessment (relationship between mother and physician) and helping alliance questionnaire (relationship between mother and case worker) at all follow up points
Zhang, Limaye & Means (2021) [47]	Between-subject design (controlled study)	**Bridges to Moms (BTM)** Collaboration between hospital and non-profit organisation that employs community-based field team to address the social determinants of health for housing-insecure pregnant women. Barriers addresses include, transportation, housing, food insecurity, personal safety, and continuity of care *Strategy:* *Resource access (transport, housing, food security, personal safety and continuity care), social support*	**Child outcomes:** Infants born to women who enrolled in intervention Bridges to Moms (BTM) for over 30 days pre-delivery (*N* = 92) and required neonatal intensive care unit had shorter stays than the comparison group (Statistical data and CI 95% are not reported) **Maternal outcomes:** Intervention group had significant postpartum clinic attendance rates and connections to primary care Women enrolled in BTM for over 30 days pre-delivery (*N* = 92) had significantly higher prenatal clinical attendance rates (Statistical data and CI 95% are not reported)

*Note*: *d* Cohen’s effect size, *p* Proportion of variance explained, *CI* Confidence interval, *NR* Not reported, *df* Degrees of freedom, *SD* Standard deviation, *t* Paired t-test, *ѱ* Unstandardized hierarchical linear growth models estimates, *F* One-factor analysis of variance

**Table 11:** Interventions of peer-based models and outcomes of included studies

**Study**	**Study design**	**Intervention details (Name of intervention, components, and intervention strategy)**	**Outcome**
Bradley et al. (2020) [39]	Within-subject design (group changes post intervention)	***Empowering Parents Empowering communities- Temporary Accommodation*** EPEC standard programme intention is based on social learning, attachment, cognitive-behavioural principles. Initial adaptations were gained by delivering standard EPEC intervention for 10 parents in the hostel followed by individual consultations. Adaptations made to design curriculum to suit temporary accommodation environment *Strategy:* Education, social support, and skill development training	**Child outcomes:** Child behaviour based on Eyberg Child Behaviour Inventory (ECBI)- Medium effect size: Cohen’s d = 0.679, 95% CI = [0.276,1.08] Concerns about my child (CAMC)- Medium effect size: Cohen’s d = 0.509, 95% CI = [0.11, 0.91] Comparison of pre-and post-test outcomes 62% of parents identified reliable improvements to childhood behaviour with 5 cases moving out of the clinical range (below 15 points on ECBI problem scale) **Maternal outcomes:** Parenting scale of hostility—Medium effect size Cohen’s d = 0.82, 95% CI = [0.39,1.24] Parental mental wellbeing = Small effect size Parenting behaviour showed overall improvement – Medium effect size Cohen’s d = 0.46, 95% CI = [0.04,0.87] Parenting scale—with reduced mean score on Parenting scale below the cut-off clinical level at follow-up. Subscale analysis identified reduction in parental hostility below clinical cut-off (2.4) and reduction parental over-reactivity from baseline No change to parenting stress, social interaction score and reduction on social support (attributed to the lack of privacy in hostel accommodation) Parental self-care and ‘the good enough parent’ were strongly endorsed topics, although some content (e.g. timeout) was deemed impractical given housing conditions
Brown et al. (2020) [40]	Within-subject design (group changes post intervention)	***Parents and Communities Together (PACT)*** Co-production of pilot study to develop meetings called “Mumspace” to facilitate social support and run health education events which were co-designed with parents, health visitors, and midwives. Some health events were co-led by parents and health professionals and some by parents only *Strategy:* Social support, (health) education, skill development training, service access	**Child outcomes: N/A** **Maternal outcomes:** Maternal mental health**—**Overall decrease in generalised anxiety and depression scores Anxiety: At the follow-up of 58 participants, there was an overall decline in GAD-7 scores at baseline and 6-month follow-up (t = 3.36 *p* = 0.001, Cohen d = 0.37) Depression: For the 61 participating mothers, the mean baseline PHQ-9 score (depression) was 7.66 (SD = 6.37), in the mild depression range. Just over one-third of the sample scored above the PHQ-9 ‘caseness’ threshold (≥ 10). At the follow-up of 58 participants, there was an overall decline in the PHQ-9 mean scores that was at baseline and follow up at 6 months 4.83 (SD = 4.15) (t = 3.78, p < 0.001) Health literacy: No changes as a whole group. Subgroup analysis there were significant improvements to health literacy for those with the mother subgroup who had low literacy at baseline (t = -3.64, *p* = 0.003) Social support: There were positive changes in the network size for advice/information (t = -3.53, *p* = 0.001), intimate interaction (t = -2.41, *p* = 0.019), and for pregnancy/childcare support (t = -2.01, *p* = 0.049), and for total network satisfaction (t = -2.06, *p* = 0.04). Reported changes in support with positive feedback, material aid, socialising, tangible assistance and total support network size were not statistically significant Parental Engagement: 93% engagement from 61 mothers with 72% fully engaged (5 or more sessions)
Gewirtz et al. (2015) [41]	Between-subject design (controlled study)	**Early Risers program** Preventative intervention in supportive housing settings for homeless families. Multicomponent: - Promoting Alternative Thinking Strategies/PATHS curriculum aimed at improving child social-emotional competence - Literacy curriculum to improve reading and comprehension skills (such as (a) reading aloud with comprehension probes, (b) vocabulary building through key words from the books, and (c) lesson-related activity sheets.) - Family/parent component e.g. Family fun nights which aimed at offering information on key child development topics with parent- child activities and a meal provided - 2nd year program included **Parenting Through Change** (PTC aims to improve five core parenting skills – teaching through encouragement, discipline, problem-solving, monitoring, and positive involvement – and is delivered using active teaching (role play and discussion) *Strategy:* Education (both child and parent), activities, skill development training (for mothers on parenting), service access (mentorship programme), social support	**Child outcomes:** *Child adjustment (externalising, internalising problems, and adaptive skills)* Children in the intervention group, Early Risers-Healthy Families Network (ER-HFN) had greater reductions in depressive symptoms relative to their control counterparts over the 2-year period (ѱ = − 2.13, *p* < .01) No different in parent reported child strengths in ER intervention group **Parenting outcomes:** Mothers in the ER-HFN interventions group had increased parenting self-efficacy than mothers in the comparison group (ѱ = .87, *p* < .05) No changes in maternal parenting practices were observed
Lee et al. (2010) [42]	Between-subject design (controlled study)	**Healthy Families Network & Early Risers Program** Partnership between non-profit organisation and family housing fund to provide affordable housing to single mothers with a history of spousal abuse, mental illness and / or substance abuse. Housing combined with early-age-target prevention program (ERP) to target children at high risk for behavioural development and poor health behaviours (see Gewirtz et al. 2015 for detail on ERP) *Strategy:* Resource access (housing), Education (both child and parent), Activities, skill development training (for mothers on parenting), service access (mentorship programme), Social support	**Child outcomes:** For child behavioural and emotional rating, no difference between children who were homeless and low-income children when teachers rated the scores Behavioural Assessment System for Children 2^nd^ Ed (BASC2): Internalising problems t = 1.11 *p* > 0.05, externalising problems, t = 0.82 *p* > 0.05 Maths and English scores for both sample groups were different but below grade level performance Academic Competence Evaluation Scales: Reading: t = 2.65, *p* < 0.001 Mathematics t = 2.92, *p* < 0.001 Maternal rating of behaviour indicated high rate of internalising and externalising child problems than low-income at-risk children Behavioural Assessment System for Children 2^nd^ Ed (BASC2): Internalising problems t = 4.31 *p* < 0.001, Externalising problems, t = 3.65 *p* < 0.001 Higher service utilisation of mental health services children experiencing homelessness compared to children who were low-income stable housing children Child Mental Health χ^2^ = 15.46 *p* < 0.001 **Maternal outcomes:** Mental health similar scores on attachment (t = 0.70, *p* > 0.05), communication (t = 1.40, *p* > 0.05), discipline practices (t = 1.51, *p* > 0.05), and involvement scales (t = 0.92, *p* > 0.05), for formerly homeless mothers in housing and low-income mothers in community setting Mothers who experienced homelessness (HFN) had worse scores on parenting confidence and relational frustration than at risk low-income community mothers (PUC) Parenting Relationship Questionnaire (PRQ): Parental confidence t = 4.85 *p* > 0.001 Relational frustration t = 6.59 *p* > 0.001 Higher service utilisation of mental health services for mothers who’ve experienced homelessness than the low-income mothers Adult Mental Health χ^2^ = 54.37 *p* < 0.001 Formerly homeless mothers experienced higher psychological distress compared to low-income mothers Mental health Brief Symptom Inventory 19 (BSI-18): Depression t = 4.32 *p* < 0.001 Anxiety t = 3.98, *p* < 0.001

*Note*: *d* Cohen’s effect size, *p* Proportion of variance explained, *CI* Confidence interval, *NR* Not reported, *df* Degrees of freedom, *SD* Standard deviation, *t* Paired t-test, *ѱ* Unstandardized hierarchical linear growth models estimates, *F* One-factor analysis of variance

#### Maternal outcomes

Nine studies investigated maternal outcomes for mothers experiencing housing insecurity (*n* = 4 maternal outcomes only, *n* = 5 both maternal and child outcomes). One of the nine studies addressed maternal outcomes by providing targeted interventions and compared mothers experiencing housing insecurity (living in the hostel) with mothers living in low-income community-based stable housing [44]. The purpose was to identify whether specific needs differ among the population groups. The nine remaining studies targeted and delivered the intervention to the same housing-insecure population either without a comparator group [39, 40] or had a service/treatment/ care-as-usual comparator group [38, 41–47]. Appendix Tables 11 and 12 outlines the comparison between comparator groups and interventions in the included studies. The effectiveness of studies is based on the impact of outcomes (health, personal and community).

Thirty maternal indicators were isolated across the 10 studies and were categorised using the O’Mara-Eves et al., (2013) extraction tool ([26] p. 191–195) which included 15 health outcomes, nine personal outcomes and six community outcomes. Maternal health outcomes indicators included parental stress, mental well-being, parenting scale (reactivity, hostility), mental health service use, depression, and anxiety (Tables 10 and 11). Nine maternal indicators from the 10 studies identified significant findings related to health outcomes for mothers experiencing housing insecurity following an intervention. Four maternal indicators identified no significant changes to health outcomes and two maternal indicators identified a negative finding post-intervention. The two negative findings were identified by Lee et al. (2010) who compared mothers living in housing insecurity to low-income mothers living in a community-based setting. The study identified mothers under housing insecurity had worse scores on parenting confidence, and relational frustration within the parent–child relationship and also experienced worse psychological distress compared to mothers in the community intervention group [42].

#### Personal outcomes

Maternal indicators for personal outcomes included self-efficacy, financial stress, transitioning out of homeless shelters, and health literacy. Eight positive findings were identified out of ten personal outcomes across the included studies (self-efficacy, financial stress, communication, family unity and transitioning out of homeless shelter and health literacy for sub-group analysis) (Table 12). Studies included overall findings which identified outcome changes when combining both the intervention and comparator groups. Brown et al., (2020) identified an overall non-significant finding for health literacy post-intervention however upon sub-group analysis, the health literacy among mothers that had low health literacy at baseline had significant improvement in health literacy at follow-up (*p* = 0.003) [40].

**Table 12:** Personal indicators of maternal outcomes across the included studies

Study	**Study design**	**Community engagement strategy**	Personal indicators	Finding
Abell et al. (2009) [38]	Between-subject design (controlled study)	Community-based case management	Communication Family relationship (unity)	Communication: *p* = 0.01 Cohen's d = 1.066 Significant difference between intervention and comparison group Family relationship (unity): *p* = 0.0135 Cohen's d = 0.952 Significant difference between intervention and comparison group
Bradley et al. (2020) [39]	Within-subject design (group changes post intervention)	Peer-led service model	N/A	N/A
Brown et al. (2020) [40]	Within-subject design (group changes post intervention)	Peer-led service model	Health literacy (overall) Health literacy (sub-group analysis)	Health literacy Overall: *p* > 0.05, 95%CI = NR, t = NR, df = NR Subgroup-analysis – Low literacy subgroup: *p* = 0.003, t = 3.64, df = 13 Intermediate literacy subgroup: *p* > 0.05, 95%CI = NR, t = NR, df = NR Adequate/high literacy subgroup: *p* > 0.05, 95%CI = NR, t = NR, df = NR
Gewirtz et al. (2015) [41]	Between-subject design (controlled study)	Peer-led service model	Parental self-efficacy	ѱ = .87, *p* < .05 95%CI = NR This suggest that mothers in the intervention group had increased in parental self- efficacy than mothers in the comparison group
Lee et al. (2010) [42]	Between-subject design (controlled study)	Peer-led service model	N/A	N/A
McWhirter (2006) [43]	Between-subject design (controlled study)	Community therapy sessions	Self-efficacy overall Self-efficacy between intervention and control groups Financial stress overall Financial stress between control groups	Self-efficacy Overall: F (1,64) = 7.68, *p* < .05, Intervention vs comparator: F (1,64) = 6.84, *p* < .05 This suggests that both the intervention and comparator group improved self-efficacy with the intervention group demonstrating stronger effect on self-efficacy than the control Financial Overall: F (1,53) = 36.35, *p* < .05, Intervention vs comparator: F (1,53) = 0.84, *p* = 0.36 This suggests that both intervention and control participants reported decreased financial stress following participation both groups
Nabors et al. (2004) [44]	Between-subject design (controlled study)	Community-based / school	N/A	N/A
Samuels et al. (2015) [45]	Between-subject design (controlled study)	Community-based case management	Transitioning out of homeless shelter	Intervention group: Average number of days till moved into stable housing: 91.25 (SD = 82.3) – Comparator group: Average number of days till moved into stable housing: 199.15 (SD = 125.4) A higher number of families in the intervention group left shelter (98%) compared with control families (84%), and the transition from shelter to housing occurred more quickly among the treatment group based on survival curve analysis
Weinreb et al. (2016) [46]	Between-subject design (controlled study)	Collaborative care model (Community-based case management)	N/A	N/A
Zhang, Limaye & Means (2021) [47]	Between-subject design (controlled study)	Community-based case management	N/A	N/A

*Note*: *d* Cohen’s effect size, *p* Proportion of variance explained, *CI* Confidence interval, *NR* Not reported, *df* Degrees of freedom, *SD* Standard deviation, *t* Paired t-test, *ѱ* Unstandardised hierarchical linear growth models estimates, *F* One-factor analysis of variance

#### Community outcomes

Three studies captured community outcomes for maternal indicators which included social support, family variables such as family conflict and bonding, and social capital (Table 13). McWhirter (2006) identified no significant changes to family variables such as a decrease in the family conflict in both group and overall analysis however, whole group analysis of social support demonstrated improved social support [43]. Brown et al. (2020) reported improvement in social support among specific categories such as the network size for advice/information (*p* = 0.001), pregnancy/childcare support (*p* = 0.049), intimate interaction (*p* = 0.019), and for total satisfaction with a social network (*p* = 0.04) [40]. Abell et al. (2009) identified improved social support in the intervention group compared to the control group [38].

**Table 13:** Community indicators of maternal outcomes across the included studies

Study	**Study design**	**Community engagement strategy**	Community indicators	Finding
Abell et al. (2009) [38]	Between-subject design (controlled study)	Community-based case management	Social support (Someone to talk to)	MD = 1.08 t = 2.23 *p* = 0.017 Cohen's d = 0.833 *r*^2^ = 0.148 Large effect size between intervention and comparison group. 14.8% of the variation of social capital "someone to talk to" was attributable to the intervention. This suggests that families in the intervention had greater social support than the comparator group
Brown et al. (2020) [40]	Within-subject design (group changes post intervention)	Peer-led service model	Social capital	Social capital pre vs. post follow up Network size for advice/information: *p* = 0.001 (t = 3.53, df = 57) Intimate interaction: *p* = 0.019 (t = 2.41, df = 57) Pregnancy/childcare support: *p* = 0.049 (t = 2.01, df = 57) Total satisfaction: *p* = 0.04 (t = 2.06, df = 57) Material aid, network size, tangible assistance was non-significant
McWhirter (2006) [43]	Between-subject design (controlled study)	Community therapy sessions	Social support (Overall and difference between groups) Family variables (Overall and difference between groups)	Social support Overall: F (1,52) = 13.81, *p* < .05 Intervention vs comparator:F (1,52) = 4.59, *p* < .05 This suggests that both groups demonstrated improved social network size and decreased social isolation. Effects were stronger among the comparator group Family variable Overall: F (1,46) = 0.45, *p* = 0.51, Intervention vs comparator: F (1,46) = 0.29, *p* = 0.59 The findings point to the potential effectiveness of both interventions but suggest that the comparator group was better in addressing social support needs of women in transition

*Note*: *d* Cohen’s effect size, *p* Proportion of variance explained, *CI* Confidence interval, *NR* Not reported, *df* Degrees of freedom, *SD* Standard deviation, *t* Paired t-test, *ѱ* Unstandardized hierarchical linear growth models estimate, *F* One-factor analysis of variance, *MD* Mean difference

#### Child outcomes

Six out of the ten studies included outcomes for children experiencing housing insecurity. One study included child outcomes only [44] and five studies included both child and maternal outcomes [38, 39, 41, 42, 47]. One of the six studies addressed child outcomes by providing targeted interventions and comparing children experiencing housing insecurity (shelter) and children from low-income backgrounds at high risk of poor behavioural outcomes and academic attainment [44]. The five remaining studies addressed child outcomes by providing interventions to the housing-insecure population with a comparator group (service/treatment/care-as-usual). The summary of child health outcomes is described in Table 14. One study used statistical modelling to analyse the influence of parenting practices and outcomes on child outcomes in the controlled study design. Gewirtz et al. (2015) conducted a hierarchical linear growth model which indicated that more effective observed parenting practices predicted high reported child strengths (ѱ = 1.58, *p* < 0.1) where for every one unit in observed parenting there where 1.58 unit increase on the interpersonal strength scale [41]. In addition, parenting self-efficacy predicated increased in child strengths (ѱ = 0.20, *p* < 0.01) [41]. Furthermore, children in the intervention group had greater reductions in depressive symptoms relative to the sample group (ѱ= −2.13, *p* < 0.01) [41].

**Table 14:** Child health outcomes from included studies

Study	**Study design**	**Community engagement strategy**	Child outcome	Finding
Abell et al. (2009) [38]	Between-subject design (controlled study)	Community-based case management	Child behaviour	*p* = 0.01, Cohen’s d = 0.75 large effect size Externalising behaviour improved significant in the intervention group than the comparison group No difference in internalising behaviour (Not reported)
Bradley et al. (2020) [39]	Within-subject design (group changes post intervention)	Peer-led service model	Concerns about my child (CAMC) Child behaviour	Child behaviour: d = 0.68, 95% CI = [0.28, 1.08] Pre- and post-test identified improved child behaviour outcomes with a medium effect size. No participants reported a deterioration in ECBI problem scores Parental rating—CAMC: d = 0.51, 95% CI = [0.11, 0.91] Prior to intervention 11 participants recorded child behaviour above clinical cut off. At post-test, 8 of out of 13 parents (62%) reported reliable improvements on the measure, including 5 cases that had moved out of the clinical cut off range
Brown et al. (2020) [40]	Within-subject design (group changes post intervention)	Peer-led service model	N/A	N/A
Gewirtz et al. (2015) [41]	Between-subject design (controlled study)	Peer-led service model	Child behaviour Child depression	Child strength (parent report): ѱ = − 0.06, *p* > 0.1 Child strength (teacher report): ѱ = − 1.58, *p* > 0.1 There was no main effect of the intervention on parent and teacher reported child strengths Child depression (parent report): ѱ = − 2.13, *p* < .01 Children in the intervention group had greater reductions in depressive symptoms relative to the control group
Lee et al. (2010) [42]	Between-subject design (controlled study)	Peer-led service model	Child behaviour (teacher report) Child behaviour (parent report) Service utilisation	Child outcomes: For child behavioural and emotional rating, no difference between children who were homeless and low-income children when teachers rated the scores Behavioural Assessment System for Children 2nd Ed (BASC2): Internalising problems t = 1.11 *p* > 0.05, externalising problems, t = 0.82 *p* > 0.05 Maths and English scores for both sample groups were different but below grade level performance Academic Competence Evaluation Scales: Reading: t = 2.65, *p* < 0.001 Mathematics t = 2.92, *p* < 0.001 Maternal rating of behaviour indicated high rate of internalising and externalising child problems than low-income at-risk children Behavioural Assessment System for Children 2nd Ed (BASC2): Internalising problems t = 4.31 *p* < 0.001, Externalising problems, t = 3.65 *p* < 0.001 Higher service utilisation of mental health services children experiencing homelessness compared to children who were low-income stable housing children Child Mental Health χ 2 = 15.46 *p* < 0.001
McWhirter (2006) [43]	Between-subject design (controlled study)	Community therapy sessions	N/A	N/A
Nabors et al. (2004) [44]	Between-subject design (controlled study)	Community-based / school	Mental health service use General health service use between two sample populations (check-up, medication, vaccination teeth cleaning, health information, medical care)	Child outcomes: 24 girls and 21 boys completed survey (mean age 7 years and 2 months) - Low income children reported seeing the same doctor on a routine bases whereas only 17% of children experiencing homelessness reported seeing a doctor regularly [Chi-squared test, χ2(1) = 8.031, *p* ≤ .01] - Children from low-income families reported receiving more medical services than children experiencing homelessness [Chi-squared test, χ2(1) = 3.994, *p* ≤ .05, t = 2.01, *p* ≤ .05] - Children experiencing homelessness reported seeing a counsellor (66.7%) than children from low-income families (7.4%) [Chi-squared test, χ2(1) = 17.696, *p* ≤ .01.] - No measurements directly on mental health / other wellbeing indicators
Samuels et al. (2015) [45]	Between-subject design (controlled study)	Community-based case management	N/A	N/A
Weinreb et al. (2016) [46]	Between-subject design (controlled study)	Collaborative care model (Community-based case management)	N/A	N/A
Zhang, Limaye & Means (2021) [47]	Between-subject design (controlled study)	Community-based case management	NICU stay	Infants born to women who enrolled in intervention Bridges to Moms (BTM) for over 30 days pre-delivery (N = 92) and required neonatal intensive care unit had shorter stays than the comparison group (Statistical data and CI 95% are not reported)

*Note*: *d* Cohen’s effect size, *p* Proportion of variance explained, *CI* Confidence interval, *NR* Not reported, *χ2* Chi-square test, *t* t-statistic *ѱ* Unstandardised hierarchical linear modelling estimates

## Discussion

The 4567 papers were identified, and post-screening 10 included studies were extracted. The majority of papers were undertaken in the UK and US after 2010 and the most common study design were observational (case–control) studies [42–44, 47]. The study quality of the papers from the UK was of high quality across all assessment components (relevancy, reliability, validity, and applicability) [39, 40]. Greater variation in the reliability of studies owing to the reporting of participant consent and ethical approval from a university board. Two papers identified moderate to high levels of fidelity to interventions with multiple sessions indicating better participant engagement [40, 41]. A large majority of papers with interventions that targeted maternal health outcomes, such as parental mental health, parenting practices and mental health service use, presented positive results (*n* = 9/16) [39, 40, 45, 46, 48]. Social support is captured as both an outcome and an intervention strategy. This reflects the dual meaning of community engagement as both a method and an end goal to facilitate improved health outcomes ([26] p. 45). Within the review, social support was the most frequent intervention strategy among the 10 papers. Social support is cited within the literature as a resource that can alleviate the stressors of homelessness experienced by mothers [49]. Non-health indicators such as social support, may provide a potential pathway for addressing maternal mental well-being. For example, the community outcomes of social support and the social network had positive results (*n* = 2) which led to an increase in network size and quality of social relationships [38, 40, 43]. Further research corroborates the review finding, where it is indicated that social support can act as a protective factor against maternal depression [50, 51]. In the context of housing insecurity, peer relationships and social support can buffer against unique stressors and address the disparity in mental health outcomes compared to the general population [49, 52].

The review demonstrated the high level of community engagement as an opportunity to increase access to statutory services by acting as a point of first entry [40]. From an institutional perspective, local authorities describe the profile of individuals experiencing housing insecurity as “hard to reach” or “hidden populations” due to their underrepresentation in the census and low engagement with local services [53, 54]. The majority of papers (*n* = 9) within the review reported a high proportion (defined as 60% or more of the sample) of non-white minority ethnic groups in the sample population of mothers and children who experience housing insecurity [38–46] (Appendix Table 14). Minority groups are also institutionally categorised as ‘hard-to-reach’ [53]; three papers within the review cited African American and Black African ethnic groups as the majority of the sample population. Broader research cites factors that contribute to being ‘hard to reach’ as a lack of awareness of available statutory services, organisational obstacles to access services (such as language barriers) and lack of cultural suitability [53, 55]. However, the review and further research support the use of peers, counsellors and community health workers as deliverers to the intervention to create relationships with marginalised communities, increase engagement and enable improvements in health outcomes [56]. The high engagement demonstrated by community engagement interventions renders the term “hard to reach” obsolete and highlights the institutional tendencies of adopting a “one size fits all” approach to health promotion [57, 58]. For instance, recent research suggests women in African communities locate mental health problems as a social issue than solely a clinical problem [40, 59, 60]. Subsequently, the review conceptualises community engagement as a cultural adaptation mechanism to improve intervention implementation by understanding contextual sensitivities of marginalised and housing insecure experiences. In addition, the incorporation of process evaluations within five identified studies provided a feedback mechanism that captured the needs of the community in order to adapt to the housing insecure context [18, 26]. For instance, the review identified an intervention that adapted an evidence-based programme to support mothers and children living in a hostel and observed improved child behaviour and parenting practice outcomes [39]. However, the participant evaluation identified concerns about privacy within hostel setting and inappropriate parenting techniques that were suggested such as 'time-out' due to the lack of space to carry out the technique in the small temporary accommodation [39]. In doing so, the review aims to operationalise a grassroots perspective that shifts the blame of being ‘hard to reach’ away from marginalised communities and allow public health practitioners to recognise the iterative process of adapting interventions to support communities in need. Furthermore, the review embraces the heterogeneity of community engagement interventions by isolating the various components, locations, and theoretical approaches. This allows policy makers and practitioners to identify potential touchpoints when engaging housing-insecure mothers, as well as highlighting the need for innovation and in-depth contextual knowledge of the population’s needs when developing interventions.

### Strengths and limitations

To the best of our knowledge, this is the first rapid review which looks at the impact of community engagement for single mothers and children experiencing housing insecurity. Consequently, the review provides insight for primary research to integrate community engagement within health interventions to better support vulnerable mothers and children. The combination of the O’Mara-Eves et al. (2013) conceptual framework, social-ecological model and maternal-child relationship provides the theoretical tools to disentangle the multivariate influences and illuminate intervention pathways for health outcomes. The basis of the rapid review captured a broad spectrum of studies from various settings such as social services, school-based environments, non-clinical health centres and shelters. The different settings reflect the potential service entry points that single mothers facing housing insecurity may encounter [61, 62].

The review aimed to rigorously search for relevant papers from extensive sources. However, there is a possibility that relevant titles may be unintentionally missed due to publication bias and the ambiguous and varied terminology surrounding community engagement. In addition, the team capacity, funding and time frame to conduct the search and screening is shorter than 12 months in comparison to other community engagement systematic review studies which may contribute to further omission and potential selection bias [63, 64]. Furthermore, due to the nature of the rapid review there is a risk of unintentional omission during screening.

Most papers were conducted in the USA and only two were in the UK. Consequently, the generalisability of results from the review is limited, especially concerning the power dynamics between institutions, governmental bodies and communities, funding structures and available resources. Therefore, public health practitioners and policymakers should interpret the results with caution by considering the contextual sensitivities in areas they aim to support.

## Conclusion

In summary, there is promising descriptive evidence of community engagement as an effective intervention towards improving the mental well-being of mothers and children living under housing insecure conditions. The review identified tentative evidence to support psychosocial maternal outcomes (self-efficacy and social support) as a potential pathway to improve maternal mental health and child outcomes; more research is needed to determine the direction and evidence of mediation pathways. In the absence of meta-analytical data, the review cannot evaluate the magnitude of the effectiveness nor make claims of causation but provides insight into modes of targeting vulnerable marginalised populations. The evidence identifies the relationship between adapted community-specific components of community engagement and greater support for maternal-child health outcomes. The review highlights the importance of process evaluations within community engagement to enable the iterative intervention adaptations to facilitate a better fit for the community rather than assuming a ‘one size fits all approach’ [26]. Consequently, the paper aims to reify the importance of understanding local knowledge for public health practitioners, policymakers, and academics within the field. Institutions working with vulnerable populations can avoid transplanting interventions from one context into another but aim to centre high levels of community engagement within the stages of public health intervention development.

### Supplementary Information

**Additional file 1:** Search Strategy.

**Additional file 2:** Quality of included studies (Critical appraisal).

**Additional file 3:** Intervention characteristics.

**Additional file 4:** Sample characteristics.

**Additional file 5:** Community Engagement Study Outcomes.

## Data Availability

The datasets used and/or analysed during the current study available from the corresponding author on reasonable request.

## References

[1] Marmot M. Fair Society, Healthy Lives: Strategic Review of Health Inequalities in England Post-2010 – the Marmot Review. In: Fair Society, Healthy Lives. Eyal N, Hurst SA, Norheim OF, Wikler D, editors. 2010. 10.1093/acprof:oso/9780199931392.003.0019.

[2] Fetzer T, Sen S, Souza P (2019). Housing insecurity, homelessness and populism: Evidence from the UK.

[3] Bess KD, Miller AL, Mehdipanah R. The effects of housing insecurity on children’s health: a scoping review. Health Promot Int. 2022.10.1093/heapro/daac006. 10.1093/heapro/daac006 35134939

[4] Stuckler D, Reeves A, Loopstra R, Karanikolos M, McKee M (2017). Austerity and health: the impact in the UK and Europe. Eur J Public Health.

[5] Fitzpatrick S, Pleace N (2012). The statutory homelessness system in England: a fair and effective rights-based model?. Hous Stud.

[6] Department for Levelling Up, Housing and Communities. National statistics - Social housing lettings in England, tenants: April 2021 to March 2022. 2023. https://www.gov.uk/government/statistics/social-housing-lettings-in-england-april-2021-to-march-2022/social-housing-lettings-in-england-tenants-april-2021-to-march-2022. Accessed 6 Sep 2023.

[7] Wilson W, Barton C (2022). Households in temporary accommodation (England).

[8] Jacobs K, Pawson H (2015). Introduction to the special edition: ‘the politics of housing policy’. Hous Stud.

[9] Edwards R (1995). Making temporary accommodation permanent: the cost for homeless families. Crit Soc Policy.

[10] Children’s Comissoner. Bleak Housing: Tackling the Crisis of Family Homelessness. England: Children’s Comissioner for England; 2019.

[11] Keilloh H. The ticking time bomb of temporary accommodation on a generation of children’s mental health. Chartered Institute of Housing. 2023. https://www.cih.org/blogs-and-articles/the-ticking-time-bomb-of-temporary-accommodation-on-a-generation-of-children-s-mental-health. Accessed 29 May 2023.

[12] Pennington J. Not-so-temporary accommodation. Shelter. 2022. https://blog.shelter.org.uk/2022/12/not-so-temporary-accommodation/. Accessed 29 May 2023.

[13] Shelter. Shelter - still living in limbo: why the use of temporary accommodation must end | Trust for London. Trust for London. 2023. https://trustforlondon.org.uk/research/shelter-still-living-in-limbo-why-the-use-of-temporary-accommodation-must-end/. Accessed 6 Jul 2023.

[14] Halpin Z (2014). Temporary accommodation in London: research findings and policy recommendations.

[15] London Councils. One in 50 Londoners homeless as ‘housing disaster unfolds in capital’. London Councils. 2023. https://beta.londoncouncils.gov.uk/news/2023/one-50-londoners-homeless-housing-disaster-unfolds-capital. Accessed 2 Aug 2023.

[16] Croft LA, Marossy A, Wilson T, Atabong A (2021). A building concern? The health needs of families in temporary accommodation. J Public Health.

[17] Rosenthal DM, Hayward A, Ucci M, Teakle A, O’Toole S, Whitaker L, et al. 576 Parental mental health and associations between living in temporary accommodation and socio-political determinants during the COVID-19 pandemic. In: British Association for Community Child Health. BMJ Publishing Group Ltd and Royal College of Paediatrics and Child Health; 2022. A71–2. 10.1136/archdischild-2022-rcpch.117.

[18] Bassuk EL, Beardslee WR (2014). Depression in homeless mothers: addressing an unrecognized public health issue. Am J Orthopsychiatry.

[19] Department for Levelling Up, Housing and Communities. Statutory Homelessness Live Tables | Statutory Homelessness in England (Table TA2). https://www.gov.uk/government/statistical-data-sets/live-tables-on-homelessness#statutory-homelessness-live-tables. Accessed 29 May 2023.

[20] NICE. Community engagement: improving health and wellbeing and reducing health inequalities | NICE Guideline. London: National Institute for Health and Care Excellence; 2016.

[21] Thomson H, Atkinson R, Petticrew M, Kearns A (2006). Do urban regeneration programmes improve public health and reduce health inequalities? A synthesis of the evidence from UK policy and practice (1980–2004). J Epidemiol Community Health.

[22] Thomson H (2008). A dose of realism for healthy urban policy: lessons from area-based initiatives in the UK. J Epidemiol Community Health.

[23] Strobl J (2000). Achieving wider participation in strategic health planning: experience from the consultation phase of Liverpool’s City Health Plan’. Health Promot Int.

[24] World Health Organisation (2002). Community participation in local health and sustainable development: approaches and techniques.

[25] Wilcox D (1994). Community participation and empowerment: putting theory into practice. RRA Notes.

[26] O’Mara-Eves A, Brunton G, McDaid D, Oliver S, Kavanagh J, Jamal F (2013). Community engagement to reduce inequalities in health: a systematic review, meta-analysis and economic analysis.

[27] Page, McKenzie, Bossuyt, Boutron, Hoffmann, Mulrow CD, et al. The PRISMA 2020 statement: an updated guideline for reporting systematic reviews. BMJ. 2021;372:n71. 10.1136/bmj.n71. 10.1136/bmj.n71 PMC8005924 33782057

[28] Philips V (2022). Searching the deep web and grey literature.

[29] Frederick TJ, Chwalek M, Hughes J, Karabanow J, Kidd S (2014). How stable is stable? defining and measuring housing stability. J Community Psychol.

[30] Swainston K, Summerbell C (2008). The effectiveness of community engagement approaches and methods for health promotion interventions.

[31] Higgins JPT, Thomas J. Cochrane Handbook for Systematic Reviews of Interventions (Wiley Cochrane Series). 2nd edition. Wiley-Blackwell. 2019.

[32] Fazel S, Geddes JR, Kushel M (2014). The health of homeless people in high-income countries: descriptive epidemiology, health consequences, and clinical and policy recommendations. Lancet.

[33] McHugh ML (2012). Interrater reliability: the kappa statistic. Biochem Med (Zagreb).

[34] Cotterill S, Knowles S, Martindale A-M, Elvey R, Howard S, Coupe N (2018). Getting messier with TIDieR: embracing context and complexity in intervention reporting. BMC Med Res Methodol.

[35] Campbell M, Katikireddi SV, Hoffmann T, Armstrong R, Waters E, Craig P (2018). TIDieR-PHP: a reporting guideline for population health and policy interventions. BMJ.

[36] Rosella LC, Pach B, Morgan S, Bowman C, Ontario Agency for Health Protection and Promotion (Public Health Ontario) (2015). Meta-tool for quality appraisal of public health evidence: PHO MetaQAT.

[37] Rosella L, Bowman C, Pach B, Morgan S, Fitzpatrick T, Goel V (2016). The development and validation of a meta-tool for quality appraisal of public health evidence: Meta Quality Appraisal Tool (MetaQAT). Public Health.

[38] Abell ML, Davey TL, Clark P, Perkins NH (2009). Community case management intervention for hard-to-place homeless families leaving emergency shelter. J Soc Distress Homeless.

[39] Bradley C, Day C, Penney C, Michelson D (2020). “Every day is hard, being outside, but you have to do it for your child”: Mixed-methods formative evaluation of a peer-led parenting intervention for homeless families. Clin Child Psychol Psychiatry.

[40] Brown J, Luderowski A, Namusisi-Riley J, Moore-Shelley I, Bolton M, Bolton D. Can a Community-Led Intervention Offering Social Support and Health Education Improve Maternal Health? A Repeated Measures Evaluation of the PACT Project Run in a Socially Deprived London Borough. Int J Environ Res Public Health. 2020;17. 10.3390/ijerph17082795. 10.3390/ijerph17082795 PMC7215628 32325635

[41] Gewirtz AH, DeGarmo DS, Lee S, Morrell N, August G (2015). Two-year outcomes of the Early Risers prevention trial with formerly homeless families residing in supportive housing. J Fam Psychol.

[42] Lee SS, August GJ, Gewirtz AH, Klimes-Dougan B, Bloomquist ML, Realmuto GM (2010). Identifying unmet mental health needs in children of formerly homeless mothers living in a supportive housing community sector of care. J Abnorm Child Psychol.

[43] McWhirter PT (2006). Community therapeutic intervention for women healing from trauma. The Journal for Specialists in Group Work.

[44] Nabors LA, Weist MD, Shugarman R, Woeste MJ, Mullet E, Rosner L (2004). Assessment, prevention, and intervention activities in a school-based program for children experiencing homelessness. Behav Modif.

[45] Samuels J, Fowler PJ, Ault-Brutus A, Tang D-I, Marcal K (2015). Time-limited case management for homeless mothers with mental health problems: effects on maternal mental health. J Soc Social Work Res.

[46] Weinreb L, Upshur CC, Fletcher-Blake D, Reed G, Frisard C. Managing depression among homeless mothers: pilot testing an adapted collaborative care intervention. Prim Care Companion CNS Disord. 2016;18. 10.4088/PCC.15m01907. 10.4088/PCC.15m01907 PMC4956430 27486545

[47] Zhang AL, Limaye N, Means RH (2021). Social determinants of health for pregnant housing-insecure women through an integrated, innovative care delivery model. J Gen Intern Med.

[48] Zhang J, Lee S-K, Piehler TF, Gewirtz AH, August GJ (2020). Bidirectional relations between parenting practices and child externalizing behaviors in formerly homeless families: a random-intercept cross-lagged panel analysis. Parent Sci Pract.

[49] Tischler V, Rademeyer A, Vostanis P (2007). Mothers experiencing homelessness: mental health, support and social care needs. Health Soc Care Community.

[50] Lefkovics E, Rigó J, Kovács I, Talabér J, Szita B, Kecskeméti A (2018). Effect of maternal depression and anxiety on mother’s perception of child and the protective role of social support. J Reprod Infant Psychol.

[51] McManus BM, Poehlmann J (2012). Maternal depression and perceived social support as predictors of cognitive function trajectories during the first 3 years of life for preterm infants in Wisconsin. Child Care Health Dev.

[52] Vostanis P, Tischler V, Cumella S, Bellerby T (2001). Mental health problems and social supports among homeless mothers and children victims of domestic and community violence. Int J Soc Psychiatry.

[53] Boag-Munroe G, Evangelou M (2012). From hard to reach to how to reach: a systematic review of the literature on hard-to-reach families. Res Pap Educ.

[54] Bristow K, Edwards S, Funnel E, Fisher L, Gask L, Dowrick C (2011). Help seeking and access to primary care for people from “Hard-to-Reach” groups with common mental health problems. Int J Family Med.

[55] Kovandžić M, Chew-Graham C, Reeve J, Edwards S, Peters S, Edge D (2011). Access to primary mental health care for hard-to-reach groups: from “silent suffering” to “making it work”. Soc Sci Med.

[56] Cyril S, Smith BJ, Possamai-Inesedy A, Renzaho AMN (2015). Exploring the role of community engagement in improving the health of disadvantaged populations: a systematic review. Glob Health Action.

[57] Wise J (2009). NICE chief criticises “one size fits all” approach to health promotion. BMJ.

[58] Hui A, Latif A, Hinsliff-Smith K, Chen T (2020). Exploring the impacts of organisational structure, policy and practice on the health inequalities of marginalised communities: Illustrative cases from the UK healthcare system. Health Policy.

[59] Brown JSL, Casey SJ, Bishop AJ, Prytys M, Whittinger N, Weinman J (2011). How black African and white British women perceive depression and help-seeking: a pilot vignette study. Int J Soc Psychiatry.

[60] Elias L, Singh A, Burgess RA (2021). In search of “community”: a critical review of community mental health services for women in African settings. Health Policy Plan.

[61] Rabiah-Mohammed F, Oudshoorn A, Forchuk C (2020). Gender and experiences of family homelessness. J Soc Distress Homelessness.

[62] Portwood SG, Shears JK, Nelson EB, Thomas ML (2015). Examining the impact of family services on homeless children. Child Fam Soc Work.

[63] Waffenschmidt S, Knelangen M, Sieben W, Bühn S, Pieper D (2019). Single screening versus conventional double screening for study selection in systematic reviews: a methodological systematic review. BMC Med Res Methodol.

[64] Shemilt I, Khan N, Park S, Thomas J (2016). Use of cost-effectiveness analysis to compare the efficiency of study identification methods in systematic reviews. Syst Rev.

