# Supplementary material for: The impact of community engagement as a public health intervention to support the mental well-being of single mothers and children living under housing insecure conditions – a rapid literature review
Source: BMC Public Health. 2023 Sep 26;23:1866. doi: 10.1186/s12889-023-16668-7 (PMC10523618; doi:10.1186/s12889-023-16668-7)
Supplement: Supplementary file 1 — Additional file 1: Search Strategy. [file 12889_2023_16668_MOESM1_ESM.docx]

Additional file 1 – Search Strategy

Table of Contents

[Appendix table 1 – Embase 2](#_Toc121825208)

[Appendix table 2 - Medline 4](#_Toc121825209)

[Appendix table 3 – Global Health 6](#_Toc121825210)

[Appendix table 4 - Child Development & Adolescent studies 7](#_Toc121825211)

[Appendix table 5 – PsychINFO 9](#_Toc121825212)

[Carrot2 Engine Search 10](#_Toc121825213)

| **Appendix table 1** – Embase | | |
| --- | --- | --- |
| **#** | **Query** | **Results from 12 May 2022** |
| 1 | exp homeless person/ or exp homelessness/ | 15,166 |
| 2 | (homeless* or hostel* or shelter* or statutory service* or evict* or crowding or overcrowd* or crowd* or public housing or 'housing tenure' or dwelling).ab. | 100,46 |
| 3 | (homeless* or hostel* or shelter* or statutory service* or evict* or crowding or overcrowd* or crowd* or public housing or 'housing tenure' or dwelling).ti. | 32,248 |
| 4 | ((temporar* or emergency or vulnerabl* or insecur* or precarious or unstabl* or short-term) adj3 (accomodat* or hous*)).ab. | 4,453 |
| 5 | ((temporar* or emergency or vulnerabl* or insecur* or precarious or unstabl* or short-term) adj3 (accomodat* or hous*)).ti. | 1,027 |
| 6 | *displacement/ | 72 |
| 7 | 1 or 2 or 3 or 4 or 5 or 6 | 116,956 |
| 8 | exp female/ or exp child/ or exp infant/ | 11,782,710 |
| 9 | exp mother/ or exp woman/ | 10,525,610 |
| 10 | (wom#n or girl* or female* or mother* or famil* or care* or maternal or antenatal or bab* or prenatal or playgroup* or birth* or child* or infant* or neonat* or newborn* or pregnan* or postnatal).ab. | 7,970,750 |
| 11 | (wom#n or girl* or female* or mother* or famil* or care* or maternal or antenatal or bab* or prenatal or playgroup* or birth* or child* or infant* or neonat* or newborn* or pregnan* or postnatal).ti. | 3,170,606 |
| 12 | 8 or 9 or 10 or 11 | 14,795,436 |
| 13 | 7 and 12 | 77,379 |
| 14 | exp Community Participation/ or exp Community-Based Participatory Research/ or exp Faith-Based Organizations/ or exp Health Promotion/ or Stakeholder Participation/ or exp Peer Group/ or exp Self-Help Groups/ or exp Group Processes/ | 171,622 |
| 15 | (commun* adj3 (engag* or organi#ing or organi* or collab* or advocacy or group* or class* or circle* or club* or committee or facilitat* or meeting* or program* or participant or stakeholder)).ab. | 81,601 |
| 16 | (commun* adj3 (engag* or organi#ing or organi* or collab* or advocacy or group* or class* or circle* or club* or committee or facilitat* or meeting* or program* or participant or stakeholder)).ti. | 12,257 |
| 17 | ((stakeholder* or community or network* or peer* or 'self help') adj3 (participat* or empower* or engag* or involv*)).ab. | 57,828 |
| 18 | community-led.ti. | 229 |
| 19 | community-led.ab. | 713 |
| 20 | 14 or 15 or 16 or 17 or 18 or 19 | 288,793 |
| 21 | exp Mental health/ or exp social support/ or exp Social capital/ | 293,457 |
| 22 | (((mental or emotional or psycho*) adj2 (health or wellbeing or well-being or ill* or disorder* or condition* or problem* or difficult*)) or (psychological* or psychiatric or psychopathology or depressi* or anxiety or suicid* or stress* or distress* or drug* or substance* or self-harm* or 'self harm*' or self-injur* or 'self injur*' or 'non-suicidal-self-injur*' or 'NSSI')).ab. | 4,740,341 |
| 23 | (((mental or emotional or psycho*) adj2 (health or wellbeing or well-being or ill* or disorder* or condition* or problem* or difficult*)) or (psychological* or psychiatric or psychopathology or depressi* or anxiety or suicid* or stress* or distress* or drug* or substance* or self-harm* or 'self harm*' or self-injur* or 'self injur*' or 'non-suicidal-self-injur*' or 'NSSI')).ti. | 1,516,827 |
| 24 | (social adj2 (support or networks or relationships or inclusion or exclusion or isolation)).ab. | 92,428 |
| 25 | (social adj2 (support or networks or relationships or inclusion or exclusion or isolation)).ti. | 18,120 |
| 26 | (self-efficacy or self-esteem or self-worth or (self and (efficacy or esteem or worth))).ab. | 113,456 |
| 27 | (self-efficacy or self-esteem or self-worth or (self and (efficacy or esteem or worth))).ti. | 15,781 |
| 28 | 21 or 22 or 23 or 24 or 25 or 26 or 27 | 5,306,676 |
| 29 | 13 and 20 and 28 | 2,379 |

| **Appendix table 2** - Medline | | | |
| --- | --- | --- | --- |
| # | Query: **Ovid MEDLINE(R) and Epub Ahead of Print, In-Process, In-Data-Review & Other Non-Indexed Citations, Daily and Versions**1946 to May 10, 2022 | **Results from 12 May 2022** |  |
| 1 | exp homeless person/ or exp homelessness/ | 10,463 |  |
| 2 | (homeless* or hostel* or shelter* or statutory service* or evict* or crowding or public housing or 'housing tenure' or dwelling).ab. | 69,309 |  |
| 3 | (homeless* or hostel* or shelter* or statutory service* or evict* or crowding or public housing or 'housing tenure' or dwelling).ti. | 24,739 |  |
| 4 | ((temporar* or emergency or vulnerabl* or insecur* or precarious or unstabl* or short-term) adj3 (accomodat* or hous*)).ab. | 3,500 |  |
| 5 | ((temporar* or emergency or vulnerabl* or insecur* or precarious or unstabl* or short-term) adj3 (accomodat* or hous*)).ti. | 871 |  |
| 6 | *displacement/ | 0 |  |
| 7 | 1 or 2 or 3 or 4 or 5 or 6 | 81,949 |  |
| 8 | exp female/ or exp child/ or exp infant/ | 10,509,494 |  |
| 9 | exp mother/ or exp woman/ | 91,654 |  |
| 10 | (wom#n or girl* or female* or mother* or famil* or care* or maternal or antenatal or bab* or prenatal or playgroup* or birth* or child* or infant* or neonat* or newborn* or pregnan* or postnatal).ab. | 5,820,017 |  |
| 11 | (wom#n or girl* or female* or mother* or famil* or care* or maternal or antenatal or bab* or prenatal or playgroup* or birth* or child* or infant* or neonat* or newborn* or pregnan* or postnatal).ti. | 2,750,779 |  |
| 12 | 8 or 9 or 10 or 11 | 12,958,623 |  |
| 13 | 7 and 12 | 53,952 |  |
| 14 | exp Community Participation/ or exp Community-Based Participatory Research/ or exp Faith-Based Organizations/ or exp Health Promotion/ or Stakeholder Participation/ or exp Peer Group/ or exp Self-Help Groups/ or exp Group Processes/ | 324,142 |  |
| 15 | (commun* adj3 (engag* or organi#ing or organi* or collab* or advocacy or group* or class* or circle* or club* or committee or facilitat* or meeting* or program* or participant or stakeholder)).ab. | 62,073 |  |
| 16 | (commun* adj3 (engag* or organi#ing or organi* or collab* or advocacy or group* or class* or circle* or club* or committee or facilitat* or meeting* or program* or participant or stakeholder)).ti. | 10,874 |  |
| 17 | ((stakeholder* or community or network* or peer* or 'self help') adj3 (participat* or empower* or engag* or involv*)).ab. | 46,688 |  |
| 18 | community-led.ti. | 180 |  |
| 19 | community-led.ab. | 591 |  |
| 20 | 14 or 15 or 16 or 17 or 18 or 19 | 411,749 |  |
| 21 | exp Mental health/ or exp social support/ or exp Social capital/ | 128,292 |  |
| 22 | (((mental or emotional or psycho*) adj2 (health or wellbeing or well-being or ill* or disorder* or condition* or problem* or difficult*)) or (psychological* or psychiatric or psychopathology or depressi* or anxiety or suicid* or stress* or distress* or drug* or substance* or self-harm* or 'self harm*' or self-injur* or 'self injur*' or 'non-suicidal-self-injur*' or 'NSSI')).ab. | 3,519,331 |  |
| 23 | (((mental or emotional or psycho*) adj2 (health or wellbeing or well-being or ill* or disorder* or condition* or problem* or difficult*)) or (psychological* or psychiatric or psychopathology or depressi* or anxiety or suicid* or stress* or distress* or drug* or substance* or self-harm* or 'self harm*' or self-injur* or 'self injur*' or 'non-suicidal-self-injur*' or 'NSSI')).ti. | 1,273,700 |  |
| 24 | (psychological* or psychiatric or psychopathology or depressi* or anxiety or suicid* or stress* or distress* or drug* or substance* or self-harm* or 'self harm*' or self-injur* or 'self injur*' or 'non-suicidal-self-injur*' or 'NSSI').ti. | 1,182,833 |  |
| 25 | (social adj2 (support or networks or relationships or inclusion or exclusion or isolation)).ab. | 74,993 |  |
| 26 | (social adj2 (support or networks or relationships or inclusion or exclusion or isolation)).ti. | 16,022 |  |
| 27 | (self-efficacy or self-esteem or self-worth or (self and (efficacy or esteem or worth))).ab. | 86,166 |  |
| 28 | (self-efficacy or self-esteem or self-worth or (self and (efficacy or esteem or worth))).ti. | 13,680 |  |
| 29 | 21 or 22 or 23 or 24 or 25 or 26 or 27 or 28 | 4,027,357 |  |
| 30 | 13 and 20 and 29 | 1,715 |  |

| **Appendix table 3** – Global Health | | |
| --- | --- | --- |
| **#** | **Query** | **Results** |
| S18 | S8 AND S12 AND S17 | 688 |
| S17 | S13 or S14 or S15 or S16 | 610,298 |
| S16 | TI (self-efficacy or self-esteem or self-worth or (self and (efficacy or esteem or worth or confidence))) OR AB (self-efficacy or self-esteem or self-worth or (self and (efficacy or esteem or worth or confidence))) | 27,044 |
| S15 | TI (social N2 (support or networks or relationships or inclusion or exclusion or isolation) OR AB (social N2 (support or networks or relationships or inclusion or exclusion or isolation) | 20,421 |
| S14 | TI (((mental or emotional or psycho*) N2 (health or wellbeing or well-being or ill* or disorder* or condition* or problem* or difficult*)) or (psychological* or psychiatric or psychopathology or depressi* or anxiety or suicid* or stress* or distress* or drug* or substance* or self-harm* or 'self harm*' or self-injur* or 'self injur*' or 'non-suicidal-self-injur*' or 'NSSI')) or AB(((mental or emotional or psycho*) N2 (health or wellbeing or well-being or ill* or disorder* or condition* or problem* or difficult*)) or (psychological* or psychiatric or psychopathology or depressi* or anxiety or suicid* or stress* or distress* or drug* or substance* or self-harm* or 'self harm*' or self-injur* or 'self injur*' or 'non-suicidal-self-injur*' or 'NSSI')) | 579,458 |
| S13 | (MH "mental health") OR (MH "social networks" OR (MH "social support") or (MH "Mental stress") | 41,818 |
| S12 | S9 or S10 or S11 | 178,088 |
| S11 | TI ((stakeholder* or community or network* or peer* or 'self help') N3 (participat* or empower* or engag* or involv*) or community-led) or AB ((stakeholder* or community or network* or peer* or 'self help') N3 (participat* or empower* or engag* or involv*) or community-led) | 18,508 |
| S10 | TI ( ((commun*) N3 (engag* or organi#ing or organi* or collab* or advocacy or involv* or group* or class* or circle* or club* or committee or facilitat* or meeting* or program* or participant or stakeholder)) ) OR AB ( ((commun*) N3 (engag* or organi#ing or organi* or collab* or advocacy or involv* or group* or class* or circle* or club* or committee or facilitat* or meeting* or program* or participant or stakeholder)) ) | 31,878 |
| S9 | (MH "sense of community") OR (MH "community involvement")OR (MH " community advocacy") OR (MH "community services") OR (MH "community health") (MH "community development")(MH "community facilities ") OR (MH "community counseling") OR DE faith based OR organizations Or DE Community mental health training or (MH "health promotion") or DE Community Mental Health Services or (MH "Stakeholders") or (MH "peer relationships") or (MH "self help") | 152,729 |
| S8 | s4 and s7 | 13,968 |
| S7 | s5 or s6 | 1,265,778 |
| S6 | TI (wom#n or girl* or female* or mother* or famil* or care* or maternal or antenatal or bab* or prenatal or playgroup* or birth* or child* or infant* or neonat* or newborn* or pregnan* or postnatal) OR AB (wom#n or girl* or female* or mother* or famil* or care* or maternal or antenatal or bab* or prenatal or playgroup* or birth* or child* or infant* or neonat* or newborn* or pregnan* or postnatal) | 1,216,137 |
| S5 | (MH "Females") OR (MH "Mothers")OR (MH "Children") OR (MH "Infants") OR (MH "Women") | 652,878 |
| S4 | s1 or s2 or s3 | 28,582 |
| S3 | TI ( ((temporar* or emergency or vulnerabl* or insecur* or precarious or unstabl* or short-term) N3 (accomodat* or hous*)) ) OR AB ( ((temporar* or emergency or vulnerabl* or insecur* or precarious or unstabl* or short-term) N3 (accomodat* or hous*)) ) | 2,923 |
| S2 | TI ( homeless* or hostel* or shelter* or “statutory services” or evict* or crowding or overcrowd* or crowd* “public housing” or “housing tenure” or dwelling) ) OR AB ( homeless* or hostel* or shelter* or “statutory services” or evict* or crowding or “public housing” or “housing tenure” or dwelling) | 21,601 |
| S1 | (MH "Homeless") OR (MH "Housing") OR (MH " Social Issues") OR (MH "Shelters") OR (MH "Homeless people") | 8,292 |

| **Appendix table 4** - Child Development & Adolescent studies | | |
| --- | --- | --- |
| **#** | **Query** | **Results** |
| S20 | S10 AND S14 AND S19 | 207 |
| S19 | s15 or s16 or s17 or s18 | 121,377 |
| S18 | TI ((self*) N3 (efficacy or esteem or confidence or worth)) OR AB((self*) N3 (efficacy or esteem or confidence or worth)) | 8,163 |
| S17 | TI (social N2 (support or networks or relationships or inclusion or exclusion or isolation) OR AB (social N2 (support or networks or relationships or inclusion or exclusion or isolation) | 8,445 |
| S16 | TI (((mental or emotional or psycho*) N2 (health or wellbeing or well-being or ill* or disorder* or condition* or problem* or difficult*)) or (psychological* or psychiatric or psychopathology or depressi* or anxiety or suicid* or stress* or distress* or drug* or substance* or self-harm* or 'self harm*' or self-injur* or 'self injur*' or 'non-suicidal-self-injur*' or 'NSSI')) or AB(((mental or emotional or psycho*) N2 (health or wellbeing or well-being or ill* or disorder* or condition* or problem* or difficult*)) or (psychological* or psychiatric or psychopathology or depressi* or anxiety or suicid* or stress* or distress* or drug* or substance* or self-harm* or 'self harm*' or self-injur* or 'self injur*' or 'non-suicidal-self-injur*' or 'NSSI')) | 104,343 |
| S15 | (MH "mental health+") OR (MH "social networks+" OR (MH "social support+") or (MH "Mental stress+") | 27,503 |
| S14 | S11 or s12 or s13 | 19,736 |
| S13 | TI ((stakeholder* or community or network* or peer* or 'self help') N3 (participat* or empower* or engag* or involv*) or community-led) or AB ((stakeholder* or community or network* or peer* or 'self help') N3 (participat* or empower* or engag* or involv*) or community-led) | 2,926 |
| S12 | TI ( ((commun*) N3 (engag* or organi#ing or organi* or collab* or advocacy or involv* or group* or class* or circle* or club* or committee or facilitat* or meeting* or program* or participant or stakeholder)) ) OR AB ( ((commun*) N3 (engag* or organi#ing or organi* or collab* or advocacy or involv* or group* or class* or circle* or club* or committee or facilitat* or meeting* or program* or participant or stakeholder)) ) | 6,094 |
| S11 | (MH "sense of community+") OR(MH "community involvement+")OR (MH " community advocacy+")OR (MH "community services+") OR (MH "community health+") (MH "community development+")(MH "community facilities+") OR (MH "community counseling+") OR DE faith based OR organizations Or DE Community mental health training or (MH "health promotion+") or DE Community Mental Health Services or (MH "Stakeholders+") or (MH "peer relationships+") or (MH "self help+") | 13,248 |
| S10 | s4 and s9 | 5,471 |
| S9 | s4 or s5 or s6 or s7 or s8 | 354,987 |
| S8 | TI(child* or adolescent* or teen* or famil* or girl* or boy*) | 184,499 |
| S7 | AB(child* or adolescent* or teen* or famil* or girl* or boy*) | 269,390 |
| S6 | TI (wom#n or girl* or female* or mother* or famil* or care* or maternal or antenatal or bab* or prenatal or playgroup* or birth* or child* or infant* or neonat* or newborn* or pregnan* or postnatal) OR AB (wom#n or girl* or female* or mother* or famil* or care* or maternal or antenatal or bab* or prenatal or playgroup* or birth* or child* or infant* or neonat* or newborn* or pregnan* or postnatal) | 315,880 |
| S5 | (MH "Females+") OR (MH "Mothers+") OR (MH "Children+") OR (MH "Infants+") OR (MH "Women+") or adolescent* or teen* | 190,908 |
| S4 | s1 or s2 or s3 | 5,471 |
| S3 | TI ( ((temporar* or emergency or vulnerabl* or insecur* or precarious or unstabl* or short-term) N3 (accomodat* or hous*)) ) OR AB ( ((temporar* or emergency or vulnerabl* or insecur* or precarious or unstabl* or short-term) N3 (accomodat* or hous*)) ) | 168 |
| S2 | TI ( homeless* or hostel* or shelter* or “statutory services” or evict* or crowding or “public housing” or “housing tenure” or dwelling) ) OR AB ( homeless* or hostel* or shelter* or “statutory services” or evict* or crowding or “public housing” or “housing tenure” or dwelling) | 2,693 |
| S1 | Homeless OR Housing OR Social Issues OR Shelters OR Homeless people | 4,536 |

| **Appendix table 5** – PsychINFO | | |
| --- | --- | --- |
| **#** | **Query** | **Results** |
| S18 | S8 AND S12 AND S17 | 1,860 |
| S17 | S13 or S14 or S15 or S16 | 1,420,333 |
| S16 | TI ((self*) N3 (efficacy or esteem or confidence or worth)) OR AB((self*) N3 (efficacy or esteem or confidence or worth)) | 97,185 |
| S15 | TI (social N2 (support or networks or relationships or inclusion or exclusion or isolation) OR AB (social N2 (support or networks or relationships or inclusion or exclusion or isolation) | 109,564 |
| S14 | TI (((mental or emotional or psycho*) N2 (health or wellbeing or well-being or ill* or disorder* or condition* or problem* or difficult*)) or (psychological* or psychiatric or psychopathology or depressi* or anxiety or suicid* or stress* or distress* or drug* or substance* or self-harm* or 'self harm*' or self-injur* or 'self injur*' or 'non-suicidal-self-injur*' or 'NSSI')) or AB(((mental or emotional or psycho*) N2 (health or wellbeing or well-being or ill* or disorder* or condition* or problem* or difficult*)) or (psychological* or psychiatric or psychopathology or depressi* or anxiety or suicid* or stress* or distress* or drug* or substance* or self-harm* or 'self harm*' or self-injur* or 'self injur*' or 'non-suicidal-self-injur*' or 'NSSI')) | 1,313,673 |
| S13 | (MM "mental health+") OR (MM "social networks+" OR (MM "social support+") or (MM "Mental stress+") | 2,661 |
| S12 | S9 or S10 or S11 | 363,902 |
| S11 | TI ((stakeholder* or community or network* or peer* or 'self help') N3 (participat* or empower* or engag* or involv*) or community-led) or AB ((stakeholder* or community or network* or peer* or 'self help') N3 (participat* or empower* or engag* or involv*) or community-led) | 36,757 |
| S10 | TI ( ((commun*) N3 (engag* or organi#ing or organi* or collab* or advocacy or involv* or group* or class* or circle* or club* or committee or facilitat* or meeting* or program* or participant or stakeholder)) ) OR AB ( ((commun*) N3 (engag* or organi#ing or organi* or collab* or advocacy or involv* or group* or class* or circle* or club* or committee or facilitat* or meeting* or program* or participant or stakeholder)) ) | 80,429 |
| S9 | (MH "sense of community+") OR(MH "community involvement+")OR (MH " community advocacy+")OR (MH "community services+") OR (MH "community health+") (MH "community development+")(MH "community facilities+") OR (MH "community counseling+") OR DE faith based OR organizations Or DE Community mental health training or (MH "health promotion+") or DE Community Mental Health Services or (MH "Stakeholders+") or (MH "peer relationships+") or (MH "self help+") | 283,263 |
| S8 | s4 and s7 | 18,562 |
| S7 | s5 or s6 | 1,715,954 |
| S6 | TI (wom#n or girl* or female* or mother* or famil* or care* or maternal or antenatal or bab* or prenatal or playgroup* or birth* or child* or infant* or neonat* or newborn* or pregnan* or postnatal) OR AB (wom#n or girl* or female* or mother* or famil* or care* or maternal or antenatal or bab* or prenatal or playgroup* or birth* or child* or infant* or neonat* or newborn* or pregnan* or postnatal) | 1,715,954 |
| S5 | (MH "Females+") OR (MH "Mothers+") OR (MH "Children+") OR (MH "Infants+") OR (MH "Women+") | 324 |
| S4 | s1 or s2 or s3 | 34,082 |
| S3 | TI ( ((temporar* or emergency or vulnerabl* or insecur* or precarious or unstabl* or short-term) N3 (accomodat* or hous*)) ) OR AB ( ((temporar* or emergency or vulnerabl* or insecur* or precarious or unstabl* or short-term) N3 (accomodat* or hous*)) ) | 1,486 |
| S2 | TI ( homeless* or hostel* or shelter* or “statutory services” or evict* or crowding or “public housing” or “housing tenure” or dwelling) ) OR AB ( homeless* or hostel* or shelter* or “statutory services” or evict* or crowding or “public housing” or “housing tenure” or dwelling) | 33,039 |
| S1 | (MM "Homeless+") OR (MM "Housing+") OR (MM " Social Issues+") OR (MM "Shelters+") OR (MM "Homeless people+") | 745 |

## Bottom of Form

## Top of Form

## Bottom of Form

## Carrot2 Engine Search

Search retrieved n=118 results: [ "homeless" OR "families" OR "mother "OR “children" OR "child" OR "mental wellbeing" OR "mental health" OR "community participation" OR "community led" OR "community organising" OR "social support"]. Figure 5 shows pie-chart diagram based on closely related clusters (n=8 not shown in diagram as they have miscellaneous clusters).


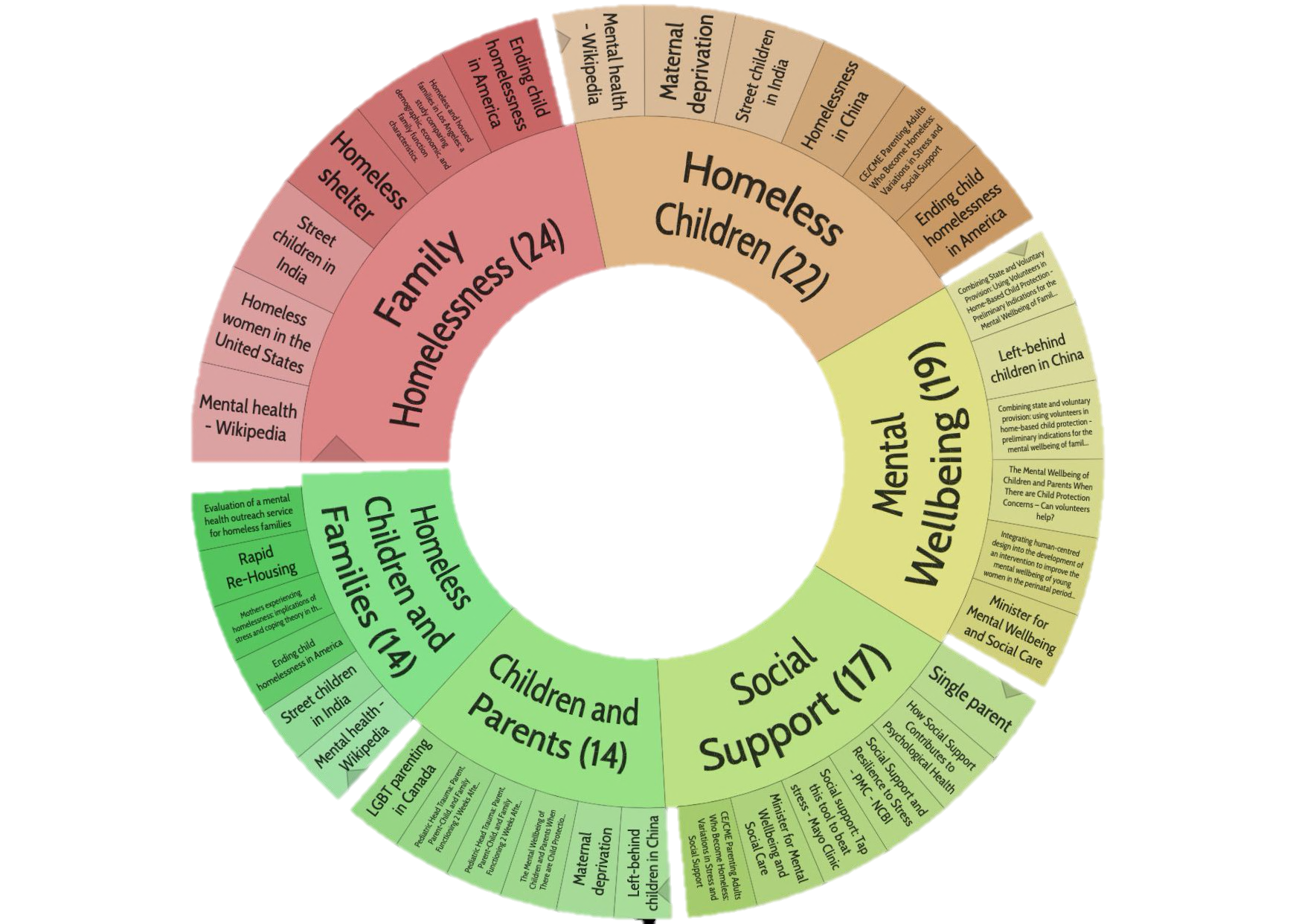


**Appendix figure 1.** Carrot2 Engine search clusters (n=6)
