# Supplementary material for: The impact of community engagement as a public health intervention to support the mental well-being of single mothers and children living under housing insecure conditions – a rapid literature review
Source: BMC Public Health. 2023 Sep 26;23:1866. doi: 10.1186/s12889-023-16668-7 (PMC10523618; doi:10.1186/s12889-023-16668-7)
Supplement: Supplementary file 2 — Additional file 2: Quality of included studies (Critical appraisal). [file 12889_2023_16668_MOESM2_ESM.docx]

Additional file 2 - Quality of included studies (Critical appraisal)

Table of Contents

[Appendix table 6 – Relevancy and reliability assessment of included studies (n=10) 2](#_Toc144135582)

[Appendix table 7 – Assessment of validity across included studies (n=10) 9](#_Toc144135583)

[Appendix table 8 – Assessment of applicability and overall score on included studies (n=10) 15](#_Toc144135584)

| **Appendix table 6 – Relevancy and reliability assessment of included studies (n=10)** | | | | | | |
| --- | --- | --- | --- | --- | --- | --- |
|  | **ASSESSMENT OF RELEVANCY** | | **ASSESSMENT OF RELIABILITY** | | | |
| **Study** | **Does the study address a topic(s) relevant to the issue under investigation? Prompting questions: Was the justification for the study clearly stated? (For example, does it address a gap in the existing literature?) Do the results of the study apply to the** | **Overall comments on relevancy?** | **a) Is the study presented clearly? Prompting questions: Is the rationale for study clearly stated, and does the study focus on a clearly defined issue? Is the conduct of the study clearly described and easy to follow? Can you identify the research design?** | **b) Are the research methodology and results clearly described? Prompting questions: Does the methodology describe the population studied, the intervention given, and the outcomes? Are all sources of information clearly identified? Are inclusion and exclusion criteria stated?** | **c) Are ethics procedures described?** Prompting questions: Was appropriate informed consent obtained? Was the study approved by an ethics review board? | **Overall comments on reliability?** |
| Abell et al. (2009) | [1]Justification is appropriately cited with relevance to UK context. Highlights the pitfalls generalising standard parenting interventions for vulnerable subgroups. [2] The results focus specifically on families living in temporary accommodation.  [3] Study population appropriate  [4] Methodologically appropriate (pre-post design primary empirical) but no comparator group. | Highly relevant | [1,2 ] Rationale for study stated. Study addresses how adapted parenting interventions can lead to improvements in wellbeing outcomes for families experiencing homelessness.  [3] Research design clearly described  [4] Results are all included  [5] Findings are clearly discussed within the appropriate context [6] Conflict of statement included  [7] Moderate level of reproducibility of the study | [1] Methodology clearly described: population (data parents living in temporary accommodation), intervention (clearly described with procedures, providers and development), outcomes (range of methods relating to objective wellbeing and evaluation of intervention). [2]Data sources are clearly described (descriptive data on participant, interview content described) [3] No inclusion and exclusion criteria defined  [4] Analytical methods described (t-distribution statistic and effect sizes)  [5] Data tables consistent with results section  [6] Methods can be reproduced based on information provided | [1] Appropriate consent obtained  [2] Ethics review board approved (Canterbury Christ Church University ethics Panel) | Highly reliable |
| Bradley et al. (2020) | [1] Justification is appropriately cited with relevance to UK context. Highlights the pitfalls generalising standard parenting interventions for vulnerable subgroups. [2] The results focus specifically on families living in temporary accommodation.  [3] Study population appropriate  [4] Methodologically appropriate (pre-post design primary empirical) but no comparator group. | Highly relevant | [1,2] Rationale for the study stated. Study addresses how adapted parenting interventions can lead to improvements in well-being outcomes for families experiencing homelessness.  [3] The research design clearly described  [4] Results are all included  [5] Findings are clearly discussed within the appropriate context [6] Conflict of statement included  [7] Moderate level of reproducibility of the study | [1] Methodology clearly described: population (data parents living in temporary accommodation), intervention (clearly described with procedures, providers and development), outcomes (range of methods relating to objective wellbeing and evaluation of intervention). [2]Data sources are clearly described (descriptive data on participant, interview content described) [3] No inclusion and exclusion criteria defined  [4] Analytical methods described (t-distribution statistic and effect sizes)  [5] Data tables consistent with results section  [6] Methods can be reproduced based on information provided | [1] Appropriate consent obtained  [2] Ethics review board approved (Canterbury Christ Church University ethics Panel) | Highly reliable |
| Brown et al. (2020) | [1,2] Justification is clearly stated (improving maternal mental health, social capital for mothers via community organisation) [3] Study population relevant (hard to reach mothers such as "shifting households") [4] Research design satisfies the methodological inclusion criteria (primary empirical studies) | Highly relevant | [1,2] Rationale clearly stated and defined issue discussed [3] Research design identified [4] All relevant results are included [5] Findings are presented and discussed within the appropriate context [6] No conflict-of-interest statement [7] Study can be reproduced based on information provided | [1] Methodology clearly described: population (data mothers such as age, number of children, first language, ethnicity), intervention (components, interventionists and environment provided), outcomes ( engagement with the intervention, health literacy, social capital, maternal mental health) [2] Data sources clearly described (descriptive data on participants) [3] No explicit mention of inclusion / exclusion criteria [4] Analytical methods (paired sample t-test) was described [5] Data tables consistent with results section [6] Methods could be reproduced based on information provided. | [1] Appropriate consent obtained from parents [2] Ethics review board approved (King's College London Research Ethics Committee) | Highly reliable |
| Gewirtz et al. (2015) | [1,2] Justification is clearly stated (evaluating intervention for high-risk children who experience homelessness for parental self-efficacy and child behavioural problems) [3] Study population relevant (homeless children and others) [4] Research design satisfies the methodological inclusion criteria (primary empirical studies) | Highly relevant | [1,2] Rationale clearly stated and defined issue extensively discussed [2] Research design identifiable [3] All relevant results are included [4] Findings are presented and discussed within the appropriate context [5] No conflict-of-interest statement [6] Study can be reproduced based on the information given | [1] Methodology clearly described: population (data on children and women such as ethnicity, age and income), intervention (components, interventionists and timeframe provided), outcomes (evaluation conducted, control variables, parental self-efficacy, parenting practices, child adjustment and attrition analyses. [2] Factors critical to the intervention are included (parental self-efficacy, practices, and child behaviour) [3] Inclusion and exclusion criteria not explicitly mentioned but limited to location of population (shelters) [4] Analytical strategy extensively discussed [5] Results reported are consistent with results section [6] Methods could be reproduced with information provided | [1] Informed consent obtained from parents  [2] Ethical approval not described | Highly reliable |

| **Appendix table 6 - continued** | | | | | | | |  |  |
| --- | --- | --- | --- | --- | --- | --- | --- | --- | --- |
| **Study** | **ASSESSMENT OF RELEVANCY** | | **ASSESSMENT OF RELIABILITY** | | | | | |  |
|  | **Does the study address a topic(s) relevant to the issue under investigation? Prompting questions: Was the justification for the study clearly stated? (For example, does it address a gap in the existing literature?) Do the results of the study apply to the** | **Overall comments on relevancy?** | | **a) Is the study presented clearly? Prompting questions: Is the rationale for study clearly stated, and does the study focus on a clearly defined issue? Is the conduct of the study clearly described and easy to follow? Can you identify the research design?** | **b) Are the research methodology and results clearly described? Prompting questions: Does the methodology describe the population studied, the intervention given, and the outcomes? Are all sources of information clearly identified? Are inclusion and exclusion criteria stated?** | **c) Are ethics procedures described? Prompting questions: Was appropriate informed consent obtained? Was the study approved by an ethics review board?** | **Overall comments on reliability?** | | |
| Lee et al. (2010) | [1,2] Justification is clearly stated (identifying psychosocial characteristics of children and assign to prevention programme) [3] Study population relevant (children and formerly homeless mothers) [4] Research design satisfies the methodological inclusion criteria (primary empirical studies) | Highly relevant | | [1,2] Rationale clearly stated and defined issue discussed [3] Research design identified [4] All relevant results are included [5] Findings are presented and discussed within the appropriate context [6] No conflict-of-interest statement [7] Study can be reproduced with the information provided | [1] Methodology adequately described: population (data on ethnicity, age, ethnicity) intervention( location of the interventions and components clearly described) outcomes (teacher reports, parental reports of child behaviour, parental mental health) [2] Factors critical to the intervention are included (parental wellbeing and child behaviour) [3] Inclusion and exclusion criteria not explicitly mentioned [4] Results reported in data tables which are consistent with described results section [5] Methods can be reproduced based on information given although intervention components lack in-depth description | [1] Informed consent obtained from parents  [2] Ethical approval not described | Highly reliable | | |
| McWhirter (2006) | [1,2] Justification is clearly stated (addressing the specific needs of homeless women at housing shelters and who need support) [3] Study population (women in family transitional shelter - childcare provided therefore mothers present) [4] Research design satisfies the methodological inclusion criteria (primary empirical studies) | Moderate relevancy | | [1,2] Rationale stated and focused on defined issue [3] Research design identified (non-experimental longitudinal study) [4] All relevant results are included [5] All findings are presented and discussed within the appropriate context [6] Study can be reproduced with information provided | [1] Methodology clearly described: population (data on women & mothers living in housing shelters), intervention (described components and delivers of intervention), outcome (social support variables, self-efficacy, financial stress, family conflict and bonding) [2] Data sources are described (participant data e.g. ethnicity and education level) [3] No explicit mention of inclusion / exclusion criteria [4] Statistical method stated (ANOVA) [5] Data tables consistent with results section [6] Methods can be reproduced based on information provided. | [1] Informed consent processes not stated  [2] Ethical approval not described | Moderate reliability: reliability limited by lack of ethical procedure. | | |
| Nabors et al. (2004) | [1,2] Justification is clearly stated (children experiencing homelessness and need to access services) [3] Study population is relevant to study [4] Research design satisfies the methodological inclusion criteria (primary empirical studies) | Highly relevant | | [1] Rationale clearly stated [2] Study described well [3] Research design identifiable (non-randomised longitudinal study) [4] All results included [5] Findings discussed in appropriate context [6] Conflict of interest not stated [7] Reproducibility provided | [1] Methodology adequately described: population **(**data on ethnicity, shelter for homeless children and their families) intervention( activities of the intervention are described with the components and delivers of intervention) outcomes (outcomes on child perceptions of mental health, service use and assessment of intervention) [2] Data sources described (brief description of data no tables) [3] No explicit inclusion / exclusion criteria (implied by children experiencing homelessness) [4] Analytical methods described (Chi-squared test) [5] No data tables [6] Methods could be reproduced based on the information provided | [1] Informed consent from parents provided  [2] Ethics review board approval not stated | Likely to be reliable but reliability is limited by ethical concerns | | |

| **Appendix table 6 - continued** | | | | | | |  |
| --- | --- | --- | --- | --- | --- | --- | --- |
| **Study** | **ASSESSMENT OF RELEVANCY** | | **ASSESSMENT OF RELIABILITY** | | | | |
|  | **Does the study address a topic(s) relevant to the issue under investigation? Prompting questions: Was the justification for the study clearly stated? (For example, does it address a gap in the existing literature?) Do the results of the study apply to the issue under consideration? How similar or different is the study population or setting to yours? Is a difference likely to matter for the issue at hand? Is the research design appropriate for the methodology you are considering? (For example, if you are considering a systematic review, you will be reviewing only certain types of publications.)** | **Overall comments on relevancy?** | **a) Is the study presented clearly? Prompting questions: Is the rationale for study clearly stated, and does the study focus on a clearly defined issue? Is the conduct of the study clearly described and easy to follow? Can you identify the research design? Are all relevant results included? Are the findings presented and discussed within the appropriate context? Is there a conflict of interest statement? Can the study be reproduced with the information provided?** | **b) Are the research methodology and results clearly described? Prompting questions: Does the methodology describe the population studied, the intervention given, and the outcomes? Are all sources of information clearly identified? Are inclusion and exclusion criteria defined? Are the statistical and/or analytical methods described? If applicable, are the results reported in data tables consistent with those described in the results section? Could the methods be reproduced based on the information provided?** | **c) Are ethics procedures described? Prompting questions: Was appropriate informed consent obtained? Was the study approved by an ethics review board?** | **Overall comments on reliability?** |  |
| Samuels et al. (2015) | [1,2] Justification is clearly stated and applies to the issue (case-management for homeless mothers with mental health problems) [3] Low generalisation concerns [4] Satisfies methodology inclusion criteria | Highly relevant | [1,2] Rationale for study stated. Study addresses how an adaptive case management model and housing can lead to reduce in acute mental health outcomes for homeless mothers.  [3] Research design clearly described  [4] Results are all included  [5] Findings are presented and discussed within the appropriate context [6] No conflict-of-interest statement provided [7] Study has high reproducibility | [1] Methodology clearly described: population (mothers with at least one child between the ages 18 months and 16 years living with them in the shelter), intervention (clearly described with procedures, providers and conditions of intervention), outcomes (range of methods relating to objective maternal mental health and fidelity to intervention) [2] Data sources clearly described (descriptive data on participants) [3] Inclusion and exclusion criteria (excludes mothers entering shelters from domestic violence due to anonymity concerns) [4] Analytical methods described (intent-to-treat analysis with time variant hierarchical linear model (HLM) [5] Data tables consistent with results section [6] Methods could be reproduced based on information provided | [1] Informed consent obtained from participants  [2] Ethical approval not described | Highly reliable |  |
| Weinreb et al. (2016) | [1,2] Study justifies the need to provide depression support for mothers who are homeless and addresses a gap in the treatment literature. [3] No issues relating to generalisability [4] Satisfies methodology inclusion criteria | Highly relevant | [1] Rationale clearly stated [2] Study described well [3] Research design identifiable (randomised control trial with trial registration number provided) [4] All results included [5] Findings discussed in appropriate context [6] Conflict of interest statement provided [7] Study can be reproduced | [1] Methodology clearly described: population (mothers with depression living in shelters), intervention (clearly described intervention of collaborative care model with procedures, providers and development), outcomes (range of methods relating to objective mental health /wellbeing, no evaluation of intervention) | [1] Consent stated but process not described  [2] Ethical approval not described | Likely to be reliable but reliability is limited by ethical concerns |  |
| Zhang, Limaye & Means (2021) | [1,2] Justification is clearly stated (housing-insecure pregnant women and new-born outcomes for case management) [3] Pregnant women may differ slightly on needs from mothers with non-new-born children [4] Satisfied methodological inclusion criteria (primary empirical studies) | Highly relevant | [1,2] Rationale clearly stated and defined issue highlighted briefly [3] Study briefly described [4] Study design not explicitly mentioned but could be identified [4] Not all results are included [5] Findings discussed briefly within housing-insecure and COVID-19 context [6] No conflict-of-interest statement [7] Study could not be reproduced | [1] Methodology poorly described: population (housing insecure pregnant mothers) intervention (Bridges to Moms community-based field team) outcomes (maternal and new-born chart data and non-profit spending although does not provide details on the chart data) [2] Data sources described [3] Inclusion/exclusion criteria not explicitly stated [4] Chance findings cannot be ruled out due to sample size [5] Unable to assess appropriateness of analysis; not described at all. [6] Methods could not be reproduced based on the information given | [1] Informed consent processes not stated  [2] Ethical approval not described | Low reliability |  |

| **Appendix table 7 – Assessment of validity across included studies (n=10)** | | | | | |
| --- | --- | --- | --- | --- | --- |
|  | **ASSESSMENT OF VALIDITY** | | | | |
| **Study** | **a) Is the study methodology appropriate for the scope of research? Prompting questions: Is the research question congruent with the study design? Does the methodology match the theory or the conceptual model? Are appropriate controls considered if applicable** | **b) Is the research methodology free from bias? Prompting questions: Were there major sources of bias with respect to: Study design? Study participants inclusion/exclusion? Measurement of exposure/outcome or important confounders/predictors? Data sources?** | **c) Are the authors’ conclusions explicit and transparent? Prompting questions: Are the results conclusive? Are the authors’ conclusions clearly derived from the results (transparent)? Are potential discrepancies discussed?** | **d) Can I be confident about the findings? Prompting questions: Are there any major methodological flaws that limit the validity of the findings? (these may have been identified in a) or b)) Are the study’s results similar to those of the existing body of** | **Overall comments on validity?** |
| Abell et al. (2009) | [1] Research design specified  [2] Methodology matches the theory of impact  [3] No controls considered (although deemed inapplicable due to low population size) [4] Statistical method appropriate given the small sample size  [5] Important theoretical factors accounted for in the analysis (homeless context, parental and child well-being) | [1] Potential bias: design (study design intensified and recruitment of participants clearly stated) participants, (no explicit inclusion/exclusion criteria - population consists of parents living in temporary accommodation); outcomes (subgroup analysis conducted).  [2] Comprehensive factors included in research (wellbeing of parent, child behaviour, evaluation of intervention to determine feasibility) [3] Results consistent within the study [4] Chance findings cannot be ruled out (no site comparisons] [5] Analyses conducted appropriately and described sufficiently. | [1] Results are conclusive  [2] Conclusions are clearly derived from results with description on impact to policy or practice  [3] Discrepancies and limitations are clearly discussed | [1] Validity of findings are high although it is limited by the pre-post measurement design. [2] Results are cited as an effective intervention for contextually relevant context. | High internal validity and external validity |
| Bradley et al. (2020) | [1] Research design specified  [2] Methodology matches the theory of impact  [3] No controls considered (although deemed inapplicable due to low population size) [4] Statistical method appropriate given small sample size  [5] Important theoretical factors accounted for in the analysis (homeless context, parental and child well-being) | [1] Potential bias: design (study design identified and recruitment of participants clearly stated) participants, (no explicit inclusion/exclusion criteria - population consists of parents living in temporary accommodation); outcomes (subgroup analysis conducted).  [2] Comprehensive factors included in research (wellbeing of parent, child behaviour, evaluation of intervention to determine feasibility) [3] Results consistent within the study [4] Chance findings cannot be ruled out (no site comparisons] [5] Analyses conducted appropriately and described sufficiently. | [1] Results are conclusive  [2] Conclusions are clearly derived from results with description on impact to policy or practice  [3] Discrepancies and limitations are clearly discussed | [1] Validity of findings are high although it is limited by the pre-post measurement design. [2] Results are cited as an effective intervention for contextually relevant context. | High internal validity and external validity |
| Brown et al. (2020) | [1] Research design specified (quasi-experimental pre-post design) [2] Methodology matches the theory of impact (intervention for maternal mental wellbeing) [3] No control group due to pre-post design structure but control variables considered [4] Statistical method appropriate [5] Important theoretical factors (maternal mental wellbeing and social capital) accounted for in the analysis | [1] Potential bias: design (study design identified - pre-post design) participants, (No explicit inclusion/ exclusion criteria but mothers in low income areas with experiences of "shifting households" and low deprivation); outcome measurement ( validated tools for parental social support and maternal mental health) [2] Factors critical to interpretation included (maternal mental health, health literacy and social support) [3] Results consistent within the study [4] Chance findings limited by study design [5] Analyses were conducted appropriately | [1] Results are conclusive [2] Authors conclusions derived from results [3] Limitations are discussed in detail | [1] High validity of findings but are limited to research design (pre-post) [2] Results are similar to existing literature | High internal validity, external validity high (may be suitable to urban context than rural) |
| Gewirtz et al. (2015) | [1] Research question congruent with design (randomised control trial) [2] Methodology matches the theory of parental child relationship under homelessness [3] Controls are considered through randomisation and isolation of variables [4] Analytical methods are appropriate for design [5] Important theoretical factors accounted for in the analysis | [1] Potential bias: design (study design identified - RCT and random selection of shelters) participants, (No explicit inclusion/ exclusion criteria but based on homeless families); outcome measurement (validated tools for parental and child outcomes) [2] Factors critical to interpretation included (parental and child outcomes) [3] Results consistent within the study [4] Chance findings are low due to the randomised structure and analytical methods [5] Analyses were conducted appropriately | [1] Results are conclusive  [2] Conclusions are clearly derived from results with description on impact to policy or practice  [3] Discrepancies and limitations are clearly discussed | [1] Validity of findings are high due to large sample size with high power  [2] Results are similar to existing literature | High internal validity, external validity high (may be suitable to urban context than rural) |

| **Appendix table 7 – continued** | | | | | |
| --- | --- | --- | --- | --- | --- |
|  | **ASSESSMENT OF VALIDITY** | | | | |
| **Study** | **a) Is the study methodology appropriate for the scope of research? Prompting questions: Is the research question congruent with the study design? Does the methodology match the theory or the conceptual model? Are appropriate controls considered if applicable** | **b) Is the research methodology free from bias? Prompting questions: Were there major sources of bias with respect to: Study design? Study participants inclusion/exclusion? Measurement of exposure/outcome or important confounders/predictors? Data sources?** | **c) Are the authors’ conclusions explicit and transparent? Prompting questions: Are the results conclusive? Are the authors’ conclusions clearly derived from the results (transparent)? Are potential discrepancies discussed?** | **d) Can I be confident about the findings? Prompting questions: Are there any major methodological flaws that limit the validity of the findings? (these may have been identified in a) or b)) Are the study’s results similar to those of the existing body of** | **Overall comments on validity?** |
| Lee et al. (2010) | [1] Research design specified and aligned with question [2] Methodology matches the theory of the conceptual model [3] Appropriate controls considered (alternative intervention for different age group) [4] Statistical method appropriate for design (t-test for difference or chi-squared test) [5] Important theoretical factors accounted for in the analysis (parental wellbeing and child behaviour) | [1] Potential bias: design (study design identified and recruitment of participants clearly stated) participants, (Inclusion criteria stated but no exclusion criteria e.g. domestic violence referrals); outcomes (parental mental health, child behaviour, family demographics and no evaluation of intervention) [2] Factors critical to the interpretation are included (parental wellbeing) [3] Results are consistent within the study [4] Chance findings cannot be ruled out due to the lack temporality in the study design [5] Analysis were carried out appropriately. | [1] Results are conclusive [2] Conclusions clearly derived from results [3] Limitations thoroughly discussed | [1] Methodological identified differences in population in the two intervention groups (formerly homeless children and at-risk children in living in community) [2] Results similar to literature | High internal validity, external validity high (may be suitable to urban context than rural) |
| McWhirter 2006 | [1] Study design specified and linked to research question although stated limitation was based on financial constraints [2] Methodology matches the theory of group therapy for subgroup population [3] Controls considered within financial constrains [4] Analytical methods appropriate given the design [5] Theoretical factors accounted for in analysis (social support, self-efficacy) | [1] Potential bias: design (study design identified; limited identification on how participants were selected or recruited), participants (no explicit inclusion / exclusion critera stated but alluded to but different population comparison i.e women in shelters and other women facing different life transition; outcome measurement (social support, financial stress, self-efficacy, familial conflict & bonding) [2] Factors critical to interpretation are included (social support variable) [3] Results consistent within the study [4] Chance findings cannot be ruled out (no pre-post design only comparison between two different groups) [5] Analysis was carried out and described | [1] Results are conclusive [2] Conclusions clearly derived from results (description of impacts on future research stated) [3] Limitations to study is discussed extensively | [1] Methodological flaws highlighted by comparator group owing to stated financial resources and 'ethical obligations to provide services'. [2] Results similar to literature and mentioned in the study | High internal validity, moderate external validity |
| Nabors et al. (2004) | [1] Research design not clearly specified but described [2] Methodology matches theory of child mental health through service use [3] Analytical method described [4] Comparator group considered from homeless children (low-income children) [5] Important theoretical factors included except for pre and post difference of mental health outcomes for children. | [1] Potential bias: design (study design identified; identification on how participants were selected or recruited), participants (no explicit inclusion / exclusion criteria stated but alluded to but different population comparison i.e. homeless children and children from low-income; outcome measurement (children's perception of mental health and prevention activities, perception of their health and mental healthcare) [2] Factors critical to interpretation are included (mental health perceptions from homeless children) [3] Results consistent within the study [4] Chance findings cannot be ruled out (no pre-post design only comparison between two different groups) [5] Analysis was carried out and described | [1] Results are conclusive [2] Conclusions derived from results [3] Potential discrepancies and delineations of results are stated. | [1] Validity of findings based are appropriate (research design, participant selection/ recruitment, inclusion / exclusion not mentioned, assessment of intervention) [2] Study results are based on limited body of literature and therefore conclusions and discussions are stated as being potentially speculative. | High internal validity, limited external validity high |
| Samuels et al. (2015) | [1] Research design/ hypothesis specified [2] Methodology clearly described; methodology matches the theory of maternal depression for homeless women [3] Service-as usual controls [4] Analytical method appropriate (controls covariates in the model and time) [5] Theoretical factors (covariates) accounted for in the analysis | [1] Potential bias: design (study design identified and recruitment of participants clearly stated) participants, (Inclusion/exclusion criteria clearly stated); outcomes (maternal mental health, housing experiences, family demographics and fidelity) [2] Important factors included in the research [3] Results are consistent within the study [4] Chance findings limited by sample size [5] Analyses conducted appropriately and described sufficiently | [1] Results are conclusive [2] Conclusions clearly derived from results (description of impacts on policy stated) [3] Limitations to study is discussed extensively | [1] Validity of findings based on methodological flaws (no baseline assessment of maternal mental health) [2] Results congruent with literature | High internal validity, external validity high (may be suitable to urban context than rural, not including domestic violence) |
| Weinreb et al. (2016) | [1] Research design specified [2] Evidence matches the theory of impact (mental wellbeing and continued care support) [3] Controls considered (service as usual comparator group rather than control) [4] Statistical method appropriate (Chi-squared test for categorical variables and t-test for normal continuous distribution and Wilcoxon rank-sum test for non-normal distribution) [5] Depression theoretical factors accounted for in the analysis (no social support measured) | [1] Potential bias: design (study design identified and recruitment of participants clearly stated) participants, (Inclusion/exclusion criteria clearly stated); outcomes (continuous and categorical data provided) [2] Important factors critical to interpretation clearly described (descriptive data on participants e.g., ethnicity, age, occupation) [3] Results consistent within the study [4] Chance findings limited by sample size [5] Analyses conducted appropriately and described sufficiently | [1] Results conclusive; limitations described [2] Conclusions clearly derived from results (impacts to depression care policy stated)  [3] Discrepancies stated (small sample size and lack of data on attrition rate profiles) | [1] Low methodological flaws, confidence limited by small sample size  [2] Outcomes for children could be considered, similar results | High internal validity, external validity high (may be suitable to urban context than rural) |
| Zhang, Limaye & Means (2021) | [1] Research design not clearly specified but were alluded to [2] Method matches theory (matching group interventions) [3] Controls considered (enrolled vs non-enrolled mothers to intervention) [4] Analytical methods not described [5] Important theoretical factors not accounted for in the analysis (psychosocial wellbeing) | [1] Potential bias: design (no study design identified; did not identify how participants were selected or recruited), participants (no explicit inclusion / exclusion criteria but different case/ control groups given); outcome measurement (no description of outcomes in tables / data provided) [2] Factors critical to interpretation were not consistently included [3] Results consistent within the study [4] Chance findings cannot be ruled out [5] Unable to assess appropriateness of analyses; not described sufficiently. | [1] Results are not conclusive; does not describe the challenges and limitations of the study [2] Conclusion derived from results but based on unpublished data [3] Discrepancies not discussed | [1] Validity of findings are low due to the methodological concerns; no assessment of effectiveness, no participant selection recruitment  [2] Study results stated that the programme (BTM) improves maternal and new-born outcomes especially under pandemic pressures | Low internal validity, limited external validity |

| **Appendix table 8 – Assessment of applicability and overall score on included studies (n=10)** | | |
| --- | --- | --- |
| **Study** | **ASSESSMENT OF APPLICABILITY** | |
|  | **How can the results be applied within the scope of public health? Prompting questions: Can the study results be interpreted and analysed within the context of public health? Are there other important public health outcomes to be considered that were not?** | **Overall comments on applicability?** |
| Abell et al. (2009) | [1] Results are likely to be interpreted within the context of public health policy  [2] Main outcomes are assessed although child outcomes could be embedded within the study  [3] Results can be applied to public health practice the applicability is limited by the methodological concerns  [4] Harms (loss of social support) and benefits (financial reimbursements) are stated [5] Relevant stakeholders (e.g. social services) were considered | Highly applicable |
| Bradley et al. (2020) | [1] Results are likely to be interpreted within the context of public health policy  [2] Main outcomes are assessed although child outcomes could be embedded within the study  [3] Results can be applied to public health practice the applicability is limited by the methodological concerns  [4] Harms (loss of social support) and benefits (financial reimbursements) are stated [5] Relevant stakeholders (e.g. social services) were considered | Highly applicable |
| Brown et al. (2020) | [1] Results highly likely to be interpreted and analysed within the context of public health and policy [2] Other important health outcomes such as parental/ child relationship outcomes not included [3] Results can be applied to public health practice based on validity and relevance [4] Harms and benefits not discussed [5] Relevant stakeholder discussions stated within community-led development of intervention | Highly applicable |
| Gewirtz et al. (2015) | [1] Results highly likely to be interpreted and analysed within the context of public health and policy [2] Other important health outcomes such as social support not included [3] Results can be applied to public health practice based on validity and relevance [4] Harms and benefits not discussed [5] Relevant stakeholder discussions not stated | Highly applicable |
| Lee et al. (2010) | [1] Results are likely to be interpreted within the context of public health policy  [2] Main outcomes are assessed [3] Results can be applied to public health practice based on relevance and validity [4] Harms and benefits not stated [5] Relevant stakeholders not explicitly mentioned | Highly applicable |
| McWhirter 2006 | [1] The results could be interpreted and analysed within the context of health policy [2] Other outcomes include objective measures of wellbeing [3] Limited to moderate level of public health practice - greater focus on psychology and group counselling [4] Harms and benefits not discussed [5] Relevant stakeholders not considered | Low applicability |
| Nabors et al. (2004) | [1] Results could be interpreted and analysed within the context of public health [2] Outcomes such as objective measures of mental wellbeing were not captured [3] Results cannot substantially be applied to public health practice, based on the validity of the article and relevance [4] No discussion of stakeholder consideration | Moderate applicability |
| Samuels et al. (2015) | [1] Results are likely to be interpreted within the context of public health policy  [2] Main outcomes are assessed although child outcomes / needs could be embedded within the study  [3] Results can be applied to public health practice based on relevance and external validity [4] Harms and benefits not discussed  [5] No explicit mention of stakeholder consideration | Highly applicable |
| Weinreb et al. (2016) | [1] Results highly likely to be interpreted and analysed within the context of public health and policy [2] Other important health outcomes such as children's need and social support not included [3] Results can be applied based on validity and relevance [3] No harms or benefits discussed [4] No explicit discussion with state holders | Highly applicable |
| Zhang, Limaye & Means (2021) | [1] Study results can be interpreted within the context of public health but no analysis [2] No explanation of maternal and new-born outcomes was elucidated beyond 'chart data' [3] Results have limited applicability to validity but has relevance to public health practice [4] Harms and benefits not discussed [5] Relevant stakeholders not mentioned for consideration | Low applicability |
