# Supplementary material for: The impact of community engagement as a public health intervention to support the mental well-being of single mothers and children living under housing insecure conditions – a rapid literature review
Source: BMC Public Health. 2023 Sep 26;23:1866. doi: 10.1186/s12889-023-16668-7 (PMC10523618; doi:10.1186/s12889-023-16668-7)
Supplement: Supplementary file 3 — Additional file 3: Intervention characteristics. [file 12889_2023_16668_MOESM3_ESM.docx]

Additional file 3 - Intervention characteristics

Table of Contents

[Appendix table 9 - Intervention strategy table of included studies (n=10) 2](#_Toc144135695)

[Appendix table 10 - TiDieR tables of included studies (n=10) 3](#_Toc144135696)

[Appendix table 11 – Comparator study overview of included studies (n=10) 12](#_Toc144135697)

[Appendix table 12 – Analysis of comparator and intervention group of included studies (n=10) 12](#_Toc144135698)

| Appendix table 9 - Intervention strategy table of included studies (n=10) | | | | | | | | | | | | | |
| --- | --- | --- | --- | --- | --- | --- | --- | --- | --- | --- | --- | --- | --- |
|  | **Intervention Strategy** | | | | | | | | | | | | |
| Study | Education | Advice | Social support | Skill development training | Activities | Resource access | Service access | Physical activity | Counselling | Clinical treatment | Medical screening | Needs assessment (not screening) | Professional training |
| Abell et al. (2009) | 0 | 0 | 1 | 0 | 0 | 1 | 1 | 0 | 0 | 0 | 0 | 0 | 0 |
| Bradley et al. (2020) | 1 | 0 | 1 | 1 | 1 | 0 | 0 | 0 | 0 | 0 | 0 | 0 | 0 |
| Brown et al. (2020) | 1 | 0 | 1 | 1 | 0 | 0 | 1 | 0 | 0 | 0 | 0 | 0 | 0 |
| Gewirtz et al. (2015) | 1 | 0 | 1 | 0 | 1 | 0 | 1 | 0 | 0 | 0 | 0 | 0 | 0 |
| Lee et al. (2010) | 1 | 0 | 1 | 1 | 1 | 1 | 1 | 0 | 0 | 0 | 0 | 0 | 0 |
| McWhirter (2006) | 0 | 0 | 1 | 0 | 0 | 0 | 0 | 0 | 1 | 0 | 0 | 0 | 0 |
| Nabors et al. (2004) | 1 | 1 | 0 | 0 | 1 | 0 | 0 | 1 | 1 | 0 | 0 | 1 | 0 |
| Samuels et al. (2015) | 0 | 0 | 1 | 0 | 0 | 1 | 1 | 0 | 0 | 1 | 0 | 1 | 0 |
| Weinreb et al. (2016) | 0 | 0 | 0 | 0 | 0 | 1 | 1 | 0 | 0 | 1 | 1 | 0 | 1 |
| Zhang, Limaye & Means (2021) | 0 | 0 | 1 | 0 | 0 | 1 | 0 | 0 | 0 | 1 | 0 | 0 | 0 |
| Frequency | 5 | 1 | 8 | 3 | 3 | 3 | 5 | 1 | 1 | 2 | 1 | 2 | 1 |
| Percentage | 12% | 2% | 20% | 7% | 7% | 7% | 12% | 2% | 2% | 5% | 2% | 5% | 2% |

| Appendix table 10 - TiDieR tables of included studies (n=10) | | | | | | |  |
| --- | --- | --- | --- | --- | --- | --- | --- |
| Study | **Context (Study population & location)** | **Theoretical conceptual approach to community participation (CP)** | **Reason for the intervention / amend to study aim** | **Intervention details (Name of intervention, components, and intervention strategy)** | **Location of community organising and length of time operating** | **Who delivered the intervention (and inclusion of specific training given)** | |
| Abell et al. (2009) | 129 participants (n=51 adults in each group) in America  Participants deemed hard to place in shelter e.g., families with male and female caregivers and single mothers with adolescent sons. (majority 67.8% were female therefore included in the study) 27 families in total  *Sample demographics* Average age of adult ranged from 19-54  72.9% African American, 5% Hispanic  Nearly 73 percent of the sample reported no income and although the median level of education received was 12 years indicating a high school graduate, more than 25 percent of the adults in the sample had fewer than 12 years of education. The average age of children in both groups (n = 78) was 10.5 years with a median of 11 years and a range from infancy to 17 years.  Most of the children in the sample were male (59%) and most identified as African-American, 92%. | Community-based case management | To explore whether families discharged from emergency shelters who receive additional case management services have improved family functioning, child-well-being and resources than families without the service | **The Community Case Management (CCM) Intervention** The CCM intervention addresses individual, family, and neighbourhood stressors that may impair family functioning and also diminish their capacity to move toward permanent housing in these vulnerable families.  *Strategy:*  Social support, service access, Resource access (food assistance), | Location: Emergency shelter site Time: 2 years | One part-time (25 hours per week) Community case manager | |
| Bradley et al. (2020) | 15 parents living in temporary accommodation in inner London. (mean age of parents = 29.21 years)  *Sample demographics* 87% were full-time carers for their children, with the remaining parents working part- or full-time. Nine parents (60%) did not have English as a first language 80% parents were from Black and minority ethnic communities  Index children (mean age = 3.6 years range = 2-9 years) | Peer-led service model to improve child behavioural difficulties, parental knowledge/practice, parental wellbeing, social support and increase accessibility, acceptability, and appropriateness of intervention | Provide targeted support to parents with housing needs:  1) improve positive parenting skills, 2) reduce child disruptive behaviours and difficulties  3) enhance coping with specific parenting challenges and stress presented by the experience of homelessness. | ***Empowering Parents Empowering communities- Temporary Accommodation***  EPEC standard programme intention is based on social learning, attachment, cognitive-behavioural principles. Initial adaptations were gained by delivering standard EPEC intervention for 10 parents in the hostel followed by individual consultations. Adaptations made to design curriculum to suit temporary accommodation environment.   *Strategy:*  Education, social support, and skill development training | Within temporary accommodation hostel in inner London 10 weeks | Peers (parents who lived in temporary accommodation, hostel, and were part of the initial formative group were trained on a peer facilitator training course) | |
| Brown et al. (2020) | 61 mothers who lived in inner London boroughs  *Sample demographics* Average age of mothers 34 years (range 22-53 years)  62% Did not have English as a first language.  55.7% Black African, 11.5% White British, 9.8% Latin American, 4.9% Asian | Peer-led service model | (1) To use community-organised and -led methods to engage women from mothers from local populations that tend to access statutory health services relatively less, and  (2) to improve maternal mental health and other health-related outcomes. | ***Parents and Communities Together (PACT)***  Co-production of pilot study to develop meetings called “Mumspace” to facilitate social support and run health education events which were co-designed with parents, health visitors, and midwives. Some health events were co-led by parents and health professionals and some by parents only.  *Strategy:* Social support, (health) education, skill development training, service access | Three local hubs: one church, one church-related centre, one community centre.  30 months | 4 paid part-time staff - community organiser, health visitor and two group leaders who were mothers from the area & volunteers for childcare. | |
| Gewirtz et al. (2015) | 161 parents (98.5% were female single-headed) and 270 children living in homeless shelters   *Sample demographics*  50% were African American, 19% were Caucasian, 20% self-identified as multiracial, and 11% other minority groups (6% Native American, 3% Hispanic, 2% Asian).  Children mean age 8.1 years | Peer-led service model | To provide evidence on prevention effects of a program for homeless families residing in family supportive housing that address parent and child outcomes. | **Early Risers program** Preventative intervention in supportive housing settings for homeless families. Multicomponent:  - Promoting Alternative Thinking Strategies/PATHS curriculum aimed at improving child social-emotional competence. - Literacy curriculum to improve reading and comprehension skills (such as (a) reading aloud with comprehension probes, (b) vocabulary building through key words from the books, and (c) lesson-related activity sheets.) - Family/parent component e.g. Family fun nights which aimed at offering information on key child development topics with parent- child activities and a meal provided. - 2nd year program included  **Parenting Through Change** (PTC aims to improve five core parenting skills – teaching through encouragement, discipline, problem-solving, monitoring, and positive involvement – and is delivered using active teaching such as role play and discussion).  *Strategy:*  Education (both child and parent), activities, skill development training (for mothers on parenting), service access (mentorship programme), social support | Location: 16 Supportive housing agencies & school Time: 2 years -  Child component two afternoons per week for 2 hours each time and a 6-week half-day summer camp held over 3 summers Parent component 14- week parent training program | Four full time Family advocates (training in PATHS and Parenting Through Change which included in person didactic presentation, coaching and guided discussion along with role playing activities). Coaching provided by ER program manager | |
| Lee et al. (2010) | One sample of children and mothers who were formerly homeless but currently living in supportive housing (HFN), and the other sample of children and mothers living in low-income, stable housing recruited via neighbourhood family resource centres (PUC) (low-income children screened based on early aggressive behaviour).  - 111 children and their mothers in supportive housing (Healthy Family Network) - 146 children in Early Risers program in PUC neighbourhood   *Sample demographics*  - HFN sample mean age of child 6.8 years - HFN sample mean age of mothers 32.6 years  - 50% African American, 21% were multiracial, 19% Caucasian, and 11% other minority groups. With both groups having similar proportions of African American families (More Caucasian and multiracial children in the HFN sample than minority group) | Peer-led service model | To evaluate the efficacy of community-based prevention services designed to reduce the risk for serious conduct problems in at-risk children | **Healthy Families Network & Early Risers Program** Partnership between non-profit organisation and family housing fund to provide affordable housing to single mothers with a history of spousal abuse, mental illness and / or substance abuse. Housing combined with early-age-target prevention program (ERP) to target children at high risk for behavioural development and poor health behaviours (see Gewirtz et al. 2015 for detail on ERP)    *Strategy:*  Resource access (housing), Education (both child and parent), Activities, skill development training (for mothers on parenting), service access (mentorship programme), Social support | Location: Housing agencies Time: NR except for 4-week questionnaire submission period. | Family advocate implementer | |
| McWhirter (2006) | 68 women  - 37 in therapy group (women living in homeless shelter for 90 until able to obtain affordable housing)  - 31 in comparison group (women experiencing major life transition e.g., job loss, domestic violence, disability, divorce, death, or spousal separation.)  *Sample demographics* Intervention group: 51.4% were European American, 27% Latina, 13.5% African American & 8.1% Native American. Median age 32 (range 22- over 65) Comparison group (alternative therapy): European American (90.3 %), and others were African American (6.5 %) and Latina (3.2 %). Median age 51 (range 22 - over 65) | Community therapy sessions | To provide mental health support that addresses the specific needs of women in housing transition (children's groups and children group therapy curriculum delivered simultaneously however the children were not assessed) | **Community-based group therapy**  Combination of cognitive behavioural and gestalt therapeutic techniques that focused around a curriculum on (a) exploring personal belief systems, especially concerning difficult experiences; (b) understanding the various forms of abuse; (c) understanding and expressing feelings; (d) recognizing healthy relationships; (e) and finding healthy ways to cope with stress.  Strategy:  Counselling, social support | Location: Community therapy session delivered in the homeless shelter.  Time:90-minute groups met weekly for 5 weeks. | Counsellor (trained in domestic violence and substance abuse) | |
| Nabors et al. (2004) | 141 homeless and high-risk children   - n=55 = children experiencing homelessness  - n= 86 low-income children who were at risk for not progressing to next grade / poor academic performance  *Sample demographics* Over 95% African American children from Baltimore | Community-based / school | To provide school-based programmes that seek to improve the physical and mental health status of children experiencing homelessness. | **School Mental Health Program & Empowerment Zone Project** - Small group prevention activities and individual counselling session for children experiencing homelessness during recreation period in afternoons. - 10 classroom sessions delivering health promotion on violence prevention, stress management, conflict resolution, risks associated with smoking and drug use, and techniques for improving emotional expression and social skills, enhancing self-esteem, using relaxation techniques, and discussed ways to be on-task, get work done, and behave appropriately in the classroom. Health promotion topics included dental and physical hygiene, learning about germs and colds and the importance of hand washing, and healthy eating and exercise habits.  *Strategy:  Education, activities (prevention activities for physical and mental health problems), counselling, advice (on enhancing self-esteem via relaxation techniques), needs assessment* | Location: Outdoor campsite  Time: 7 weeks from early July to Late August 1999. | Health professionals, counsellor | |
| Samuels et al. (2015) | 210 Homeless mothers from family homeless services in a county outside New York City. Children aged between 18 months to 16 years.  - 97 in Family Critical Time Intervention  - 113 Service-as-usual control   *Sample demographics* (No significant difference in demographic characteristics between treatment and control group) 85% identified as African American, Latino, or other ethnic minority (contrasted with 71.3% Caucasian population of the country from Census data). Mothers in late 20s or early 30s living with 3 children (SD =1.6) younger than 18 years. Average age of child was 9 years old (SD= 5) | Community-based case management | To provide psychological and parenting support interventions to mothers experiencing homelessness during accommodation transitions and increased community service support.  Designed to (a) strengthen family members’ long-term ties to the services they need,  (b) heal and strengthen maternal relationships with extended families and friends, and  (c) provide emotional and practical support during the critical time of transition from homelessness to stable housing in the community.  FCTI focuses on the relationship between the case manager and mother that progresses through the 9-month period. | **Family Critical Time Intervention protocol:** Community based case management under three phases: *Transition to Community, Try-Out & Transfer to Care*  - Intervention group received continuous case-management services from single worker with CTI training, - Managers had low-caseloads (<12) in comparison to service as usual (50:1 with high turnover rate among caseworkers) - Low threshold for housing readiness for intervention group than service-as-usual meaning families had more immediate access to transitional housing than service as usual group  Strategy: *Social support, Service (employment, child support, family children's services, medical/home care and temporary financial services) and resource access (housing)*  *support mothers with children for 9-month period as they transition from homeless shelters to affordable housing* | Location: Homeless shelter count Time: Nov 2001 till Feb 2004 | Case manager (FCTI trained case manager) | |
| Weinreb et al. (2016) | 67 women with depression (PHQ-9 score ≥ 10) were randomly selected from two primary care clinics in family residence shelters in Queens and Bronx, New York.  - 42 enrolled in intervention shelter clinic  - 25 enrolled in control shelter clinic  *Sample demographics* Women were on average 36 years of age (intervention: 35.2 years, usual care: 38 years); 52.2% had been homeless before. 81% in intervention group were non-white and 76% in usual care group were non-white | Adaptation of collaborative care model focused on engagement and increasing patient self-management | Need to provide mental health support to mothers experiencing homelessness due to vulnerability to depression | ***Integrated Care Model for Homeless Mothers*** Collaborative care model adapted to the needs of homeless mothers which included engagement interview with mothers via care manager, providing basic needs (food stamps, clothing for children, obtaining public assistance) and addressing mental health comorbidities  *Strategy:* Medical screening**,** *Professional training for staff members and access to resources and services (e.g., food stamps, clothing, diapers, obtaining public assistance)* | Location: Primary care clinics based in family residence shelters in Queens and Bronx, New York Time: Weekly/ Biweekly outreach conducted for 6-8 weeks followed by check-ins for 6 months | Health professional Case manager (social worker)  For intervention group 4 hours of training given to all staff and 20 hours to care manager who is master's level trained mental health clinician.  2-hour team training provided over 18 moths | |
| Zhang, Limaye & Means (2021) | 267 housing-insecure women  - 134 in intervention (Bridges to Mom enrolled) - 133 who did not enrol in intervention (control)  *Sample demographics* No information on demographics | Community-based case management | To target the social determinants of health for housing-insecure pregnant women. | **Bridges to Moms (BTM)**  Collaboration between hospital and non-profit organisation that employs community-based field team to address the social determinants of health for housing-insecure pregnant women. Barriers addresses include, transportation, housing, food insecurity, personal safety, and continuity of care.  *Strategy:  Resource access (transport, housing, food security, personal safety and continuity care), social support* | Location: Hospital and community Time: 2017 to 2019. BTM enrolment for over 30 days pre-delivery. | Nurse Community health worker | |

| Appendix table 11 – Comparator study overview of included studies (n=10) | | | | | | | | | | | | |
| --- | --- | --- | --- | --- | --- | --- | --- | --- | --- | --- | --- | --- |
| Criteria | Characteristics | No. studies | Abell et al. (2009) | Bradley et al. (2020) | Weinreb et al. (2016) | Samuels et al. (2015) | Zhang, Limaye & Means (2021) | Nabors et al. (2004) | McWhirter (2006) | Gewirtz et al. (2015) | Lee et al. (2010) | Brown et al. (2020) |
| Comparator |  |  |  |  |  |  |  |  |  |  |  |  |
|  | No comparator | 2 |  | 1 |  |  |  |  |  |  |  | 1 |
|  | Comparator (service-as-usual, different sample group) | 8 | 1 |  | 1 | 1 | 1 | 1 | 1 | 1 | 1 |  |
| Type of comparator | Service/treatment/care as usual | 5 | 1 |  | 1 | 1 | 1 |  |  | 1 |  |  |
|  | Alternative comparable group | 3 |  |  |  |  |  | 1 | 1 |  | 1 |  |

| Appendix table 12 – Analysis of comparator and intervention group of included studies (n=10) | | |
| --- | --- | --- |
| Study | **Comparator group** | **Intervention group** |
| Abell et al. (2009) | Service-as-usual N=51 No CCM services or CCM case manager | N=51 Community Case Management (CCM) intervention upon leaving the shelter. CCM Services included referral, advocacy, support and assistance with family problem-solving & decision making |
| Bradley et al. (2020) | N/A (Pre-post design) | Empowering Parents Empowering communities- Temporary Accommodation  N=15  EPEC standard programme intention is based on social learning, attachment, cognitive-behavioural principles. Initial adaptations were gained by delivering standard EPEC intervention for 10 parents in the hostel followed by individual consultations. Adaptations made to design curriculum to suit temporary accommodation environment. |
| Brown et al. (2020) | N/A (Pre-post design) | Parents and Communities Together (PACT) N=61 |
| Gewirtz et al. (2015) | Service-as-usual N=80 parents and n=126 children No ERI programme at site  No further explanation given on control conditions. | N= 53 parents and n=104 children Early Risers Intervention – 2-year programme  Child received after school programmes  Family support services (case management)  Family Fun nights (ran quarterly) Parenting Through Change – parenting programme |
| Lee et al. (2010) | Service-as-usual (Pillsbury United Community) N=146 children Sample of children and mothers living in low-income, stable housing recruited via neighbourhood family resource centres (low-income children screened based on early aggressive behaviour). | Healthy Family Network  N=111 children  One sample of children and mothers who were formerly homeless but currently living in supportive housing. |
| McWhirter (2006) | Comparable group N=31  Women experiencing major life transition | N=37 Community-based group therapy Women living in homeless shelter until able to obtain affordable housing |
| Nabors et al. (2004) | Comparable group  N= 86 low-income children at risk of poor academic performance | School Mental Health Program & Empowerment Zone Project  N=55 homeless youths  Participated in small group activities |
| Samuels et al. (2015) | Service as usual N=113 Assessment of needs over 2-week period whilst staying in hotel. Screening of medical, mental health, substance abuse. Interviews on clinical and non-clinical assessments e.g., pathways to homelessness, housing history etc.  Assessment centre between 30-45 days before moving to shelters managed by non-profit agencies.  Shelter sites include screening e.g., substance abuse, rehabilitation, childcare, mental health assessment, but were sparsely furnished and overcrowded.  Housing readiness training provided with independent living plans | Family Critical Time Intervention  N=97  9-month Case management model containing three phases of 3 months each.   - Phase 1 – Transition to community (case manager meets family needs and creates links to community resources and service provision for mental health, substance abuse, childcare employment - Phase 2 – Try out (Develop trust between case manager and family to provide secure links and maximise the mothers’ strengths to secure and maintain stable housing - Phase 3 – Transfer to Care (case manager reduces contact and uses previously established community care to meet needs) |
| Weinreb et al. (2016) | Service as usual  N= 25  Appointment with primary care physician (PCP) who gave usual care such as antidepressant medication and recommendation for psychotherapy.  Received general case management services such as obtaining public benefits and linking to community resources & children’s educational needs. | Integrated Care Model for Homeless Mothers  N=42 Collaborative care model for leadership engagement and proactive outreach by case managers and clinical decision-making tools, patient self-management.  Depression care manager received additional training and the PCP on collaborating with the care manager to implement outreach, regular symptom monitoring.  Collaborative care model included: (1) engagement interview at the beginning of treatment  (2) Care manager addressing basic needs and children’s needs (3) Addressing concomitant mental health comorbidities |
| Zhang, Limaye & Means (2021) | Service as usual N=133  Usual prenatal care. Care not specified. | Bridges to Mom collaboration between Hospital and Non-profit  N=134  Addresses barriers for housing-insecure pregnant women such as transportation, housing, food insecurity, personal safety and continuity of care.  Assigned a nurse and community health worker throughout pregnancy and postpartum |
