# Supplementary material for: The impact of community engagement as a public health intervention to support the mental well-being of single mothers and children living under housing insecure conditions – a rapid literature review
Source: BMC Public Health. 2023 Sep 26;23:1866. doi: 10.1186/s12889-023-16668-7 (PMC10523618; doi:10.1186/s12889-023-16668-7)
Supplement: Supplementary file 4 — Additional file 4: Sample characteristics. [file 12889_2023_16668_MOESM4_ESM.docx]

Additional file 4 - Sample characteristics

Table of Contents

[Appendix table 13 - Full table of summary characteristics from Table 2 with accompanying included studies (n=10) 2](#_Toc144135983)

[Appendix table 14 - Population characteristics using Keywording extraction tool [1 p.181-189] 4](#_Toc144135984)

[Keywording Extraction Tool [1 p.181-189] 6](#_Toc144135985)

| **Appendix table 13 -** Full table of summary characteristics from Table 2 with accompanying included studies (n=10) | | | | | | | | | | | | |
| --- | --- | --- | --- | --- | --- | --- | --- | --- | --- | --- | --- | --- |
| Criteria | Characteristics | No. studies | Abell et al. (2009) | Bradley et al. (2020) | Weinreb et al. (2016) | Samuels et al. (2015) | Zhang, Limaye & Means (2021) | Nabors et al. (2004) | McWhirter (2006) | Gewirtz et al. (2015) | Lee et al (2010) | Brown et al. (2020) |
| Year | 2001-2010 | 4 | 1 |  |  |  |  | 1 | 1 |  | 1 |  |
|  | After 2010 | 6 |  | 1 | 1 | 1 | 1 |  |  | 1 |  | 1 |
| Country | US | 8 | 1 |  | 1 | 1 | 1 | 1 | 1 | 1 | 1 |  |
|  | UK | 2 |  | 1 |  |  |  |  |  |  |  | 1 |
| Population | Mothers experiencing housing insecurity / homelessness only | 4 |  |  | 1 | 1 |  |  | 1 |  |  | 1 |
|  | Children experiencing housing insecurity / homelessness only | 1 |  |  |  |  |  | 1 |  |  |  |  |
|  | Both | 5 | 1 | 1 |  |  | 1 |  |  | 1 | 1 |  |
| Study design | Randomised control trial | 3 |  |  | 1 | 1 |  |  |  | 1 |  |  |
|  | Nonrandomised control trial | 0 |  |  |  |  |  |  |  |  |  |  |
|  | Quasi-experimental | 3 | 1 | 1 |  |  |  |  |  |  |  | 1 |
|  | Observational (case-control) | 4 |  |  |  |  | 1 | 1 | 1 |  | 1 |  |
| Comparator |  |  |  |  |  |  |  |  |  |  |  |  |
|  | No comparator | 2 |  | 1 |  |  |  |  |  |  |  | 1 |
|  | Comparator (service-as-usual, different sample group) | 8 | 1 |  | 1 | 1 | 1 | 1 | 1 | 1 | 1 |  |
| Type of comparator | Service/treatment/care as usual | 5 | 1 |  | 1 | 1 | 1 |  |  | 1 |  |  |
|  | Alternative comparable group | 3 |  |  |  |  |  | 1 | 1 |  | 1 |  |
|  | | | | | | | | | | | | |

|  | | | | | | | | | | | | |
| --- | --- | --- | --- | --- | --- | --- | --- | --- | --- | --- | --- | --- |
| Criteria | Characteristics | No. studies | Abell et al. (2009) | Bradley et al. (2020) | Weinreb et al. (2016) | Samuels et al. (2015) | Zhang, Limaye & Means (2021) | Nabors et al. (2004) | McWhirter (2006) | Gewirtz et al. (2015) | Lee et al (2010) | Brown et al. (2020) |
|  |  |  |  |  |  |  |  |  |  |  |  |  |
| Maternal outcomes | Community outcomes: Social support, social capital | 3 | 1 |  |  |  |  |  | 1 |  |  | 1 |
|  | Health outcomes (mental wellbeing, mental health service use) | 7 |  | 1 | 1 | 1 | 1 |  |  | 1 | 1 | 1 |
|  | Personal outcomes: Self-efficacy, self-confidence, self-esteem | 4 |  |  |  | 1 |  |  | 1 | 1 |  | 1 |
| Child outcomes related to mental wellbeing | Health outcomes (child behaviours, mental health service use) | 6 | 1 | 1 |  |  | 1 | 1 |  | 1 | 1 |  |
| Number of outcome measurements | Pre- and post-intervention | 3 |  | 1 |  |  |  |  | 1 |  |  | 1 |
|  | One further timepoint | 0 |  |  |  |  |  |  |  |  |  |  |
|  | Two further timepoints | 3 |  |  | 1 | 1 |  |  |  | 1 |  |  |
|  | Post intervention only | 3 |  |  |  |  | 1 | 1 |  |  | 1 |  |
| Length of follow-up period | |  |  |  |  |  |  |  |  |  |  |  |
|  | 1 month | 1 |  |  |  |  |  |  |  |  | 1 |  |
|  | 1.5 months | 1 |  |  |  |  |  |  | 1 |  |  |  |
|  | 2.5 months | 1 |  | 1 |  |  |  |  |  |  |  |  |
|  | 3 months | 2 |  |  | 1 | 1 |  |  |  |  |  |  |
|  | 6 months | 2 |  |  |  | 1 |  |  |  |  |  | 1 |
|  | 9 months | 1 |  |  | 1 |  |  |  |  |  |  |  |
|  | 12 months | 1 |  |  |  |  |  |  |  | 1 |  |  |
|  | 15 months | 1 |  |  | 1 |  |  |  |  |  |  |  |
|  | 24 months | 1 |  |  |  |  |  |  |  | 1 |  |  |
|  | No information provided in study | 2 |  |  |  |  | 1 | 1 |  |  |  |  |

| **Appendix table 14 -** Population characteristics using Keywording extraction tool [1 p.181-189] | | | | | | | | | |
| --- | --- | --- | --- | --- | --- | --- | --- | --- | --- |
| Study | How are child/maternal mental outcomes addressed? | Ethnicity | SEP (income, benefits, deprived area classification) | Employment | Education | Place of residence (rural/ urban / housing characteristics) | Gender | Age | Marital status |
| Abell et al. (2009) | Targeted at and delivered to PROGRESS-Plus population | 72.9% Adults African American 92% Children African American 5% Hispanic adult 5% Other adult racial group | Majority (73%) of the sample had no income | NR | Median level of education was 12 years including high school graduate, More than 25% of adults had less than 12 years of education | Urban, Homeless shelters | Majority female adults | Median age 35 years,  Mean age 34.29 years (range 19 to 54 years) 10.5 years for children (range infant - 17 years) | NR |
| Bradley et al. (2020) | Targeted at and delivered to PROGRESS-Plus population | 80% Black and minority ethnic communities 60% English not first language | Deprivation in inner London borough with high rates of socioeconomic deprivation | 13% In full-time or part time work | NR | Urban, housing characteristics (temporary accommodation in hostel) | Majority female | NR | NR |
| Brown et al. (2020) | Targeted at and delivered to PROGRESS-Plus population | 55.7% Black African 11.5% White British 11.5% White any other background 9.8% Latin American 4.9% Asian | NR | 36.1% unemployed  9.7% unskilled 23% Partially skilled | 41% Undergraduate degree 16.4% BTEC/NVQ equivalent | Urban | All female | Maternal age 34 years (22-53 years) | 50.8% Married or living with someone 32.8% Single |
| Gewirtz et al. (2015) | Targeted at and delivered to PROGRESS-Plus population | 50% African American 19% Caucasian 20% Multi-racial  6% Native American 3% Hispanic  2% Asian | $10,371.59 Average annual income | NR | On average parents were high school grads or equivalent with (M = 11.98 years of education) | Urban, supportive housing | Majority female | N/A maternal age  Child age mean 8.10 years | NR |
| Lee et al. (2010) | Targeted and comparison within a PROGRESS-Plus category | Intervention group 50% African American 19% Caucasian 21% Multiracial  11% Other minority groups | Majority had annual income less than $20,000 | NR | Intervention group (homeless mothers) 11.93 years of education on average | Unclear | All female | Maternal age for intervention (32.55)  Child age average 6.77 years | NR |
| McWhirter (2006) | Targeted at and delivered to PROGRESS-Plus population | Intervention group 51.4% European American 27% Hispanic / Latina 13.5% African American 8.1% Native American | NR | NR | 37.8% graduated high school | Unclear | All female | Intervention group: Median age 32 (22 to over 65) | NR |
| Nabors et al. (2004) | Targeted and comparison within a PROGRESS-Plus category | Over 95% African American | NR | NR | NR | Urban | 24 girls & 21 boys | Mean age 7 years and 2 months | NR |
| Samuels et al. (2015) | Targeted at and delivered to PROGRESS-Plus population | 49% African American 18% Hispanic / Latina | $250 Monthly income | 15% Currently employed | 19% graduated high school or equivalent degree | Urban, Homeless shelters | All female | 32.5 (Average maternal age) 9 (average child age) | 27% Married or cohabiting |
| Weinreb et al. (2016) | Targeted at and delivered to PROGRESS-Plus population | 47.6% Black  42.9% Hispanic / Latina | $756 Median income | 12.2 % Currently employed | 33.3% graduated high school | Urban, Homeless shelters | Majority female | Average age: 36 years old – overall  35.2 years - intervention 38 years- control | NR |
| Zhang, Limaye & Means (2021) | Targeted at and delivered to PROGRESS-Plus population | NR | NR | NR | NR | Urban | All female | NR | NR |
| NR = Not reported, PROGRESS Plus is an acronym denoting place of residence, race/ethnicity/culture/language, occupation, gender/sex, religion, education, socioeconomic status, and social capital. The Plus refers to time-dependent relationships, personal characteristics associated with discrimination and features of relationships [2] | | | | | | | | | |

## **Keywording Extraction Tool [1 p.181-189]**

O’Mara-Eves A, Brunton G, McDaid D, et al. Community engagement to reduce inequalities in health: a systematic review, meta-analysis and economic analysis. Southampton (UK): NIHR Journals Library, 2013.

1. In which country does the study take place?
2. Name of public health intervention (specify if applicable)
3. Age group(s) of participants
4. Sex of participants
5. What is the main PROGRESS-Plus characteristic of the sample/population? (ethnicity, socioeconomic status, occupation, education, place of residence, sexual orientation, social capital, gender, religion, age, marital status/family composition, disability)
   - - - No main PROGRESS-Plus group (not mentioned in title and abstract)
       - Multiple equally important characteristics (specify) If there is more than one main characteristic that cannot be distinguished (i.e., are equally focused upon), then use this code and specify
6. How are health inequalities addressed?

- Targeted at or delivered to specific PROGRESS-Plus group Targeted or delivered to a specific PROGRESS-Plus population. Usually, the disadvantaged group will constitute the majority or entire sample of participants
- Universal (aimed at the whole group population, not on the basis of individual needs/risks) Interventions that target the general public or a whole population group that has not been identified on the basis of individual risk or needs. The PROGRESS-Plus group will usually be identified through subgroup analyses

1. What was the primary health issue being addressed?
2. What health issues were incorporated into the intervention content/ delivery? This can be stated anywhere in the text.
3. To what extent was community engagement integral to the public health intervention?
   - - - Completely community engagement (Completely = all components of intervention delivered through)
       - Mostly community engagement (mostly = 50% or more of components of intervention were delivered through CE)
       - Little community engagement (CE; little = less than 50% of intervention components were delivered through CE)
       - Omit if no community engagement
4. Was the community explicitly involved in identifying the health problem/need?
5. Intervention site
6. Person(s) delivering the PH intervention (intervenors)

- Community member *From the community, not employed. Does not include those labelled as ‘peer’*
- Community worker *Employed as a worker in a community. Not necessarily from the community*
- Computer
- Counsellor
- Health professional (e.g., GP, dietitian, nurse)
- Health promotion practitioner
- Lawyer
- Parent
- Peer *Peers defined as people sharing the same age group (e.g., schools peers) or health behaviour/risk/ condition or similar in key aspects (e.g., race/ethnicity, SES, geographic location)*
- Psychologist
- Religious leader (e.g., parson, priest)

1. Is this a multicomponent intervention? (Yes/ No)

13a) Intervention type (State all that apply)

- Activities (e.g., community fairs/fetes)
- Advice One-to-one communication consisting of directional guidance, recommendations, or suggestions. Element of tailoring
- Education Information only, e.g., information leaflets, curriculum
- Skill development/training Skills are practised or modelled, e.g., condom use, assertiveness skills
- Counselling (based on the psychological needs of the individual)
- Social support
- Environmental modification e.g., improvement in housing conditions, provision of healthier school meals
- Immunisation
- Incentives e.g., participants received money, tickets for a prize draw, credits for a course
- Professional training For interventions targeted at changing professional practice including those involving medical students, etc.
- Physical activity, exercise
- Rehabilitation
- Resource access e.g., condom distribution
- Risk assessment (not medical screening) e.g., environmental assessment for risk of falling in older people
- Role modelling, role playing
- Screening. For medical screening, e.g., breast screening
- Service access e.g., establishing a drop-in centre, extended opening times of a family planning clinic, referral to a service
- Clinical treatment
- Biofeedback e.g., feedback on a personal basis of increased levels of carbon monoxide in one’s breath as a prevention strategy for smoking
- Other

1. For each aspect of an initiative (design, delivery, evaluation) what was the level of community engagement?
   - Design/planning
     - - Leading - Responsibility and decision-making authority reside with the community members
       - Collaborating - Community members have shared responsibility and authority for design with others
       - Consulted (piloting of intervention is included here if there is a feedback mechanism) Community members are asked about design/planning, but authority and responsibility lie outside the community. Includes focus groups
       - Informed - Community members told what’s going to happen to them
       - Other
       - Not involved/unclear
       - Delivery
       - Leading - Responsibility and decision-making authority reside with the community members
       - Collaborating - Community members have shared responsibility and authority for design with others
       - Consulted (piloting of intervention is included here if there is a feedback mechanism) Community members are asked about design/planning, but authority and responsibility lie outside the community. Includes focus groups
       - Informed - Community members told what’s going to happen to them
       - Other
       - Not involved/unclear
     - Evaluation
       - Leading - Responsibility and decision-making authority reside with the community members
       - Collaborating - Community members have shared responsibility and authority for design with others
       - Consulted (piloting of intervention is included here if there is a feedback mechanism) Community members are asked about design/planning, but authority and responsibility lie outside the community. Includes focus groups
       - Informed - Community members told what’s going to happen to them
       - Other
       - Not involved/unclear
2. Label/s for community engagement strategy

- Community action/support; community mobilisation/involvement/engagement/participation
- Community organisations – developing new and existing services
- Community partnership; community coalitions; community task force Also includes forum; committee; advisory group
- Any peer involvement, e.g., peer counselling, peer education, peer leaders, peer leadership, role models, peer support Peers defined as people sharing the same age group (e.g. schools peers) or health behaviour/risk/condition or similar in key aspects (e.g. race/ethnicity). NOT promotoras
- Non-peer health advocacy (e.g., lay health, community health workers) For members of the community that are NOT peers of the target participants, where ‘peer’ is defined as sharing the same age group or health risk/condition or similar in key aspects (e.g. race/ethnicity). NOT promotoras
- Promotora (explicit use of term) Must use specific ‘promotora’ label
- ‘Outreach’ programme (explicit use of term)
- Social networks (explicit use of term)
- Volunteering/volunteers (explicit use of term)
- Other community engagement strategy
- No clear community engagement strategy

1. Was training provided to the engages? (Yes/No/ Not stated or unclear)
2. Were outcomes measured for the engages (people participating) in community engagement (Yes/No)
3. What categories of outcomes were reported?

- Health outcomes, e.g., behaviours, knowledge, attitudes. Can include health service use outcomes such as number of visits to a clinic
- Personal outcomes e.g., empowerment, self-esteem, efficacy, skills
- Community outcomes, e.g., capacity building, social capital or inclusion
- Process outcomes e.g., acceptability, appropriateness
- Cost or resource use data, or cost-effectiveness
- Not stated/unclear
- Other

1. Is there anything within this study that is not reflected in our conceptual framework?Top of FormBottom of Form

Bibliography

1. O’Mara-Eves A, Brunton G, McDaid D, Oliver S, Kavanagh J, Jamal F, et al. Community engagement to reduce inequalities in health: a systematic review, meta-analysis and economic analysis. Southampton (UK): NIHR Journals Library; 2013.

2. O’Neill J, Tabish H, Welch V, Petticrew M, Pottie K, Clarke M, et al. Applying an equity lens to interventions: using PROGRESS ensures consideration of socially stratifying factors to illuminate inequities in health. J Clin Epidemiol. 2014;67:56–64. doi:10.1016/j.jclinepi.2013.08.005.
