# Supplementary material for: The impact of community engagement as a public health intervention to support the mental well-being of single mothers and children living under housing insecure conditions – a rapid literature review
Source: BMC Public Health. 2023 Sep 26;23:1866. doi: 10.1186/s12889-023-16668-7 (PMC10523618; doi:10.1186/s12889-023-16668-7)
Supplement: Supplementary file 5 — Additional file 5: Community Engagement Study Outcomes. [file 12889_2023_16668_MOESM5_ESM.docx]

Additional file 5 – Community Engagement Study Outcomes

Table of Contents

[Appendix table 15 – Community engagement strategy and outcomes based on the included studies (n=10) 2](#_Toc144136023)

[Appendix table 16 – Labels of community engagement strategies of included studies (n=10) 12](#_Toc144136024)

| **Appendix table 15 –** Community engagement strategy and outcomes based on the included studies (n=10) | | |
| --- | --- | --- |
| **Study** | **Community engagement strategy** | **Outcome** |
| **Abell et al. (2009)** | Community-based case management | **Child outcomes** Children externalising behaviour improved in the intervention group compared to the control group (p=0.01, Cohen's d=0.75 large effect size). No difference in internalising behaviours (Cohen’s d not reported). Family outcomes:  Social support increased in intervention group  Large effect size d=0.833, p=0.017, 95% CI = NR  Improved family relationship over control group: Large effect size d=0.952, p=0.0135, 95% CI = NR)  Communication subscale  Large effect size d=1.066, p=0.01, 95% CI = NR  Unity dimension  Large effect size d= 0.952 p=0.0135, 95% CI = NR |
| **Bradley et al. (2020)** | Peer-led service model | **Child outcomes:** Child behaviour based on Eyberg Child Behaviour Inventory (ECBI)- Medium effect size:  Cohen’s d=0.679, 95% CI =[0.276,1.08]  Concerns about my child (CAMC)- Medium effect size: Cohen’s d = 0.509, 95% CI =[0.11, 0.91]  Comparison of pre-and post-test outcomes 62% of parents identified reliable improvements to childhood behaviour with 5 cases moving out of the clinical range (below 15 points on ECBI problem scale).  **Maternal outcomes:** Parenting scale of hostility - Medium effect size Cohen’s d=0.82, 95% CI =[0.39,1.24] Parental mental wellbeing = Small effect size  Parenting behaviour showed overall improvement – Medium effect size  Cohen’s d=0.46, 95% CI=[0.04,0.87]  Parenting scale - with reduced mean score on Parenting scale below the cut-off clinical level at follow-up. Subscale analysis identified reduction in parental hostility below clinical cut-off (2.4) and reduction parental over-reactivity from baseline.  No change to parenting stress, social interaction score and reduction on social support (attributed to the lack of privacy in hostel accommodation). Parental self-care and ‘the good enough parent’ were strongly endorsed topics, although some content (e.g. timeout) was deemed impractical given housing conditions. |
| **Brown et al. (2020)** | Peer-led service model | **Child outcomes: N/A  Maternal outcomes:** Maternal mental health **-** Overall decrease in generalised anxiety and depression scores  Anxiety: At the follow-up of 58 participants, there was an overall decline in GAD-7 scores at baseline and 6-month follow-up (t=3.36 p=0.001, Cohen d=0.37).  Depression: For the 61 participating mothers, the mean baseline PHQ-9 score (depression) was 7.66 (SD=6.37), in the mild depression range. Just over one-third of the sample scored above the PHQ-9 ‘caseness’ threshold (≥10). At the follow-up of 58 participants, there was an overall decline in the PHQ-9 mean scores that was at baseline and follow up at 6 months 4.83 (SD=4.15) (t=3.78, p<0.001).   Health literacy: No changes as a whole group. Subgroup analysis there were significant **i**mprovements to health literacy for those with the mother subgroup who had low literacy at baseline (t=-3.64, p=0.003)  Social support: There were positive changes in the network size for advice/information (t= -3.53, p=0.001),, intimate interaction (t= -2.41, p=0.019), and for pregnancy/childcare support (t= -2.01, p=0.049), and for total network satisfaction (t= -2.06, p=0.04).   Parental Engagement - 93% engagement from 61 mothers with 72% fully engaged (5 or more sessions). |
| **Gewirtz et al. (2015)** | Peer-led service model | **Child outcomes:** *Child adjustment (externalising, internalising problems, and adaptive skills)* Children in the intervention group, Early Risers-Healthy Families Network (ER-HFN) had greater reductions in depressive symptoms relative to their control counterparts over the 2-year period (ѱ = −2.13, p <.01).  No different in parent reported child strengths in ER intervention group. **Parenting outcomes:** Mothers in the ER-HFN interventions group had increased parenting self-efficacy than mothers in the comparison group (ѱ = .87, p <.05).  No changes in maternal parenting practices were observed. |
| **Lee et al. (2010)** | Peer-led service model | **Child outcomes:** For child behavioural and emotional rating, no difference between children who were homeless and low-income children when teachers rated the scores.  Behavioural Assessment System for Children 2^nd^ Ed (BASC2):  Internalising problems t=1.11 p>0.05, externalising problems, t = 0.82 p>0.05  Maths and English scores for both sample groups were different but below grade level performance Academic Competence Evaluation Scales:  Reading: t = 2.65, p<0.001  Mathematics t = 2.92, p<0.001  Maternal rating of behaviour indicated high rate of internalising and externalising child problems than low-income at-risk children.  Behavioural Assessment System for Children 2^nd^ Ed (BASC2):  Internalising problems t=4.31 p<0.001, Externalising problems, t = 3.65 p<0.001  Higher service utilisation of mental health services children experiencing homelessness compared to chilren who were low-income stable housing children.  Child Mental Health χ ^2^ = 15.46 p<0.001  **Maternal outcomes:** Mental health similar scores on attachment (t=0.70, p>0.05), communication (t=1.40, p>0.05), discipline practices (t=1.51, p>0.05), and involvement scales (t=0.92, p>0.05), for formerly homeless mothers in housing and low-income mothers in community setting. Mothers who experienced homelessness (HFN) had worse scores on parenting confidence and relational frustration than at risk low-income community mothers (PUC).  Parenting Relationship Questionnaire (PRQ): Parental confidence t=4.85 p>0.001 Relational frustration t=6.59 p>0.001  Higher service utilisation of mental health services for mothers who’ve experienced homelessness than the low-income mothers.  Adult Mental Health χ ^2^ = 54.37 p<0.001  Formerly homeless mothers experienced higher psychological distress compared to low-income mothers  Mental health Brief Symptom Inventory 19 (BSI-18):  Depression t=4.32 p<0.001  Anxiety t=3.98, p<0.001 |
| **McWhirter (2006)** | Community therapy sessions | **Child outcomes: N/A** **Maternal outcomes:** - Social support variables: Overall increase between both groups in social network size and social isolation with a significant increase among the comparison group (women in transition). Calculated using one-factor ANOVA Social Network Size, F (1, 53) ¼ .02, p > .01, Social Isolation, F (1,66) ¼ .43, p > .01 - Self-efficacy greater increase in group among women who were living in homeless shelters (intervention) than women in transition group (alternative comparison group i.e. individual educational mentorship group) [Self-efficacy F (1,65) ¼ .49, p > .01.] - Decreases in financial stress following participation in both groups Financial Stress, [F (1,53) ¼ .19, p > .01] -Family conflict [Family Conflict, F (1,64) ¼ .00, p > .01] - No effect on family bonding and / or family conflict for either interventions [Family Bonding F (1,59) ¼ .26, p > .01] |
| **Nabors et al. (2004)** | Community-based / school | **Child outcomes:** 24 girls and 21 boys completed survey (mean age 7 years and 2 months) - Low income children reported seeing the same doctor on a routine bases whereas only 17% of children experiencing homelessness reported seeing a doctor regularly [Chi-squared test, χ^2^(1) = 8.031, p ≤ .01]  - Children from low-income families reported receiving more medical services than children experiencing homelessness [Chi-squared test, χ^2^(1) = 3.994, p ≤ .05, t= 2.01, p ≤ .05 ] - Children experiencing homelessness reported seeing a counsellor (66.7%) than children from low-income families (7.4%) [Chi-squared test, χ^2^(1)= 17.696, p ≤ .01.]  - No measurements directly on mental health / other wellbeing indicators **Maternal outcomes**: N/A |
| **Samuels et al. (2015)** | Community-based case management | **Child outcomes: N/A  Maternal outcomes:** Between baseline and 15-month assessments, mothers reported a 9-point decrease, on average, in standard Global Severity Index scores. The decrease brought the majority of mothers into the normal range of mental health relative to the general adult population. No significant difference in mental health and rate of decline between the intervention, Family Critical Time Intervention (FCTI) and control group.  At 9 month follow up mothers with elevated symptoms declined by 35% in intervention and 35% in control group.  FCTI intervention group transitioned into permanent housing faster than service-as-usual group however this did not correspond to improvements in maternal mental health. |
| **Weinreb et al. (2016)** | Collaborative care model (Community-based case management) | **Child outcomes: N/A**  **Maternal outcomes:**  Overall significant deduction in depression score for all women by the end of the follow up ( At 6 months follow up, women in the intervention group had a high per proportion of over 50% improvement in depression symptoms (intervention: 30%, usual care: 5.9%, P = .07).  Women in the intervention group also had significantly more primary care physician (PCP) and care manager visits at both follow ups:  *3-month*  PCP visits being two or more - intervention: 74.3%, usual care: 53.3%, P = .009; Care manager visits being two or more – intervention: 91.4%, usual care: 26.7%, P < .001  *6-month*  PCP visits being two or more - intervention: 46.7%, usual care: 23.5%, P = .003.  Care manager visits being two or more – intervention 70%, usual care: 17.7%, P = .001,  Medication prescription for depression – intervention: 73.3%, usual care: 5.9%, P ≤ .001.  No difference between intervention and usual care groups when assessing anxiety, mental or physical health functioning, patient reactions assessment (relationship between mother and physician) and helping alliance questionnaire (relationship between mother and case worker) at all follow up points. |
| **Zhang, Limaye & Means (2021)** | Community-based case management | **Child outcomes:** Infants born to women who enrolled in intervention Bridges to Moms (BTM) for over 30 days pre-delivery (N = 92) and required neonatal intensive care unit had shorter stays than the comparison group.  (Statistical data and CI 95% are not reported)   **Maternal outcomes:**  Intervention group had significant postpartum clinic attendance rates and connections to primary care.  Women enrolled in BTM for over 30 days pre-delivery (N = 92) had significantly higher prenatal clinical attendance rates. (Statistical data and CI 95% are not reported) |

| **Appendix table 16** – Labels of community engagement strategies of included studies (n=10) | | | | | | |
| --- | --- | --- | --- | --- | --- | --- |
| **Study** | **Community action/support, community mobilisation/involvement/ engagement/participation** | **Community organisations – developing new and existing** | **Community coalition, community partnership, community task force** | **Any peer strategy (e.g., peer counselling, peer education, peer leaders, peer leadership, role models, peer support)** | **Non-peer health advocacy (e.g., lay health workers, community health)** | **Social networks (explicit mention of social network)** |
| Abell et al (2009) | 0 | 0 | 0 | 0 | 1 | 0 |
| Bradley et al. (2020) | 0 | 0 | 0 | 1 | 0 | 0 |
| Brown et al. (2020) | 1 | 1 | 1 | 1 | 0 | 1 |
| Gewirtz et al. (2015) | 0 | 1 | 0 | 0 | 1 | 0 |
| Lee et al. (2010) | 0 | 1 | 0 | 0 | 0 | 0 |
| McWhirter (2006) | 0 | 1 | 0 | 1 | 0 | 1 |
| Nabors et al. (2004) | 0 | 1 | 0 | 0 | 0 | 0 |
| Samuels et al. (2015) | 0 | 1 | 0 | 0 | 0 | 0 |
| Weinreb et al. (2016) | 0 | 1 | 0 | 0 | 0 | 0 |
| Zhang, Limaye & Means (2021) | 0 | 1 | 0 | 0 | 0 | 0 |
| Total | 1 | 8 | 1 | 3 | 2 | 2 |
